# Immune characterization of pre-clinical murine models of neuroblastoma

**DOI:** 10.1038/s41598-020-73695-9

**Published:** 2020-10-07

**Authors:** Emily R. Webb, Silvia Lanati, Carol Wareham, Alistair Easton, Stuart N. Dunn, Tatyana Inzhelevskaya, Freja M. Sadler, Sonya James, Margaret Ashton-Key, Mark S. Cragg, Stephen A. Beers, Juliet C. Gray

**Affiliations:** 1 Antibody and Vaccine Group, Centre for Cancer Immunology, University of Southampton Faculty of Medicine, Southampton General Hospital (MP127), Tremona Road, Southampton, Hampshire SO16 6YD UK; 2 Cellular Pathology, University Hospitals Southampton NHS Foundation Trust, Southampton, SO16 6YD UK; 3 Present Address: Edinburgh Cancer Research Centre, Institute of Genetics and Molecular Medicine, University of Edinburgh, Western General Hospital, Edinburgh, EH4 2XU UK; 4 Present Address: Department of Oncology, University of Oxford, Old Road Campus Research Building, Roosevelt Drive, Oxford, OX3 7DQ UK

**Keywords:** Cancer, Immunology

## Abstract

Immunotherapy offers a potentially less toxic, more tumor-specific treatment for neuroblastoma than conventional cytotoxic therapies. Accurate and reproducible immune competent preclinical models are key to understanding mechanisms of action, interactions with other therapies and mechanisms of resistance to immunotherapy. Here we characterized the tumor and splenic microenvironment of two syngeneic subcutaneous (NXS2 and 9464D), and a spontaneous transgenic (TH-MYCN) murine model of neuroblastoma, comparing histological features and immune infiltrates to previously published data on human neuroblastoma. Histological sections of frozen tissues were stained by immunohistochemistry and immunofluorescence for immune cell markers and tumor architecture. Tissues were dissociated by enzymatic digestion, stained with panels of antibodies to detect and quantify cancer cells, along with lymphocytic and myeloid infiltration by flow cytometry. Finally, we tested TH-MYCN mice as a feasible model for immunotherapy, using prior treatment with cyclophosphamide to create a therapeutic window of minimal residual disease to favor host immune development. Immune infiltration differed significantly between all the models. TH-MYCN tumors were found to resemble immune infiltration in human tumors more closely than the subcutaneous models, alongside similar GD2 and MHC class I expression. Finally, TH-MYCN transgenic mice were administered cyclophosphamide alone or in combination with an anti-GD2 or anti-4-1BB monoclonal antibody, which resulted in increase in survival in both combination therapies. The TH-MYCN transgenic mouse is a promising in vivo model for testing immunotherapy compounds and combination therapy in a preclinical setting.

## Introduction

Neuroblastoma is one of the commonest childhood malignancies, accounting for 8% of pediatric cancers, and 15% of pediatric cancer deaths. It is an embryonal tumor, arising from progenitor cells of the sympathetic nervous system, most commonly in the adrenal glands^1^. Over 50% of patients with neuroblastoma are considered to have ‘high risk’ disease, because of adverse prognostic features such as amplification of the MYCN oncogene or metastatic spread. The outcome for these patients is poor (< 50% long term survival), despite intensive multi-modal therapies^1–4^. Immunotherapy is a potentially attractive alternative or additional treatment for these patients. A number of tumor antigens have been identified and endogenous anti-tumor immune responses are documented^5,6^. The majority of clinical immunotherapies for neuroblastoma have focused on targeting the disialoganglioside GD2, expressed on virtually all neuroblastomas^7,8^. Anti-GD2 mAbs have been shown to improve outcome in first line treatment for high risk neuroblastoma, and are now considered a key component of treatment^9,10^.

Despite the improvement in outcome achieved with anti-GD2 mAbs, the many children with high-risk neuroblastoma still ultimately relapse and die from their disease^11,12^. Improving the efficacy of anti-GD2 antibody therapy, as well as developing novel immunotherapies, is a key focus of efforts to improving survival. Many different immunotherapeutic approaches have shown pre-clinical efficacy, but relatively few have progressed after clinical trials. Pre-clinical modeling is essential in order to compare and prioritize different therapies, to understand mechanisms of action and resistance, and to identify potential biomarkers. Furthermore, it is likely that maximal benefit will be achieved by using immunotherapies in combination. The number of potential combinational therapies is vast, and careful pre-clinical assessment is vital to rationally guided trial design, particularly given the small potential patient population.

Assessment of most immunotherapies requires immunocompetent in vivo models. Ideally these should be comparable in immunogenicity to human neuroblastoma, with similar endogenous anti-tumor immune responses and regulatory mechanisms. The number of such neuroblastoma models is limited. Immunocompetent models used include injected syngeneic cell lines, genetically modified murine models which develop spontaneous tumors, and cell lines derived from these. By far the majority of pre-clinical immunotherapies for neuroblastoma have been tested using syngeneic cell lines. More recently the TH-MYCN transgenic murine model has been used, with a limited number of reports describing pre-clinical immunotherapies tested using either spontaneous tumors arising in these mice, or in which cell lines established from the spontaneous tumors are injected in to wild type mice^13–17^.

TH-MYCN transgenic mice over-express MYC-N under the control of the tyrosine hydroxylase promoter and spontaneously develop aggressive tumors, closely resembling human neuroblastoma in location, histology, biology and cytogenetic abnormalities, although it is perhaps limited by the apparent lack of spontaneous metastasis^18^. Homozygous mice develop tumors at a young age, potentially before immunological maturity has been achieved. The use of heterozygous mice, which develop tumors after a longer latency, may allow for immunological competence to be developed prior to experimental testing of new therapies. 9464D is an immortalized tumor cell line derived from a spontaneous neuroblastoma arising in a TH-MYCN transgenic mouse on the C57BL/6 background^13,14^. The NSX2 cell line was developed from a subline of the NX-31T28 hybrid cell line of C1300 NB cell line and dorsal root ganglion cells and has been widely used as an immunocompetent neuroblastoma model in AJ mice^19^.

Here, we provide comprehensive analyzes of the immunological signatures of these pre-clinical mouse models and compare them with reported clinical data regarding human neuroblastoma. We focus on the TH-MYCN spontaneous tumor model, comparing this to the widely used subcutaneous models. Our results underlined the histological heterogeneity of TH-MYCN tumors, and the low expression of MHC Class-I in GD2^+^ cells in both TH-MYCN and 9464D tumors. The presence of tumor infiltrating lymphocytes (TIL) and tumor associated macrophages (TAM) in the tumor microenvironment of the models was compared. Finally, we exploited the potential of creating a therapeutic window to study antibody-based immunotherapy in the TH-MYCN model by treating mice with sub-curative doses of cyclophosphamide, showing its suitability for pre-clinical immunotherapy studies. Although there are caveats with the TH-MYCN model, such as a lack of metastasis reflected in attempts at generating a more metastatic model by engineering in a caspase-8 deficiency^20^, the histological structure, anatomical location and spontaneity of the TH-MYCN tumors, suggest this model could be considered a good translational immunocompetent murine model which can be utilized for preclinical immunotherapy evaluations for high-risk (MYCN amplified) neuroblastoma.

## Results

### Growth and survival kinetics of the three murine neuroblastoma models

**Figure 1:**
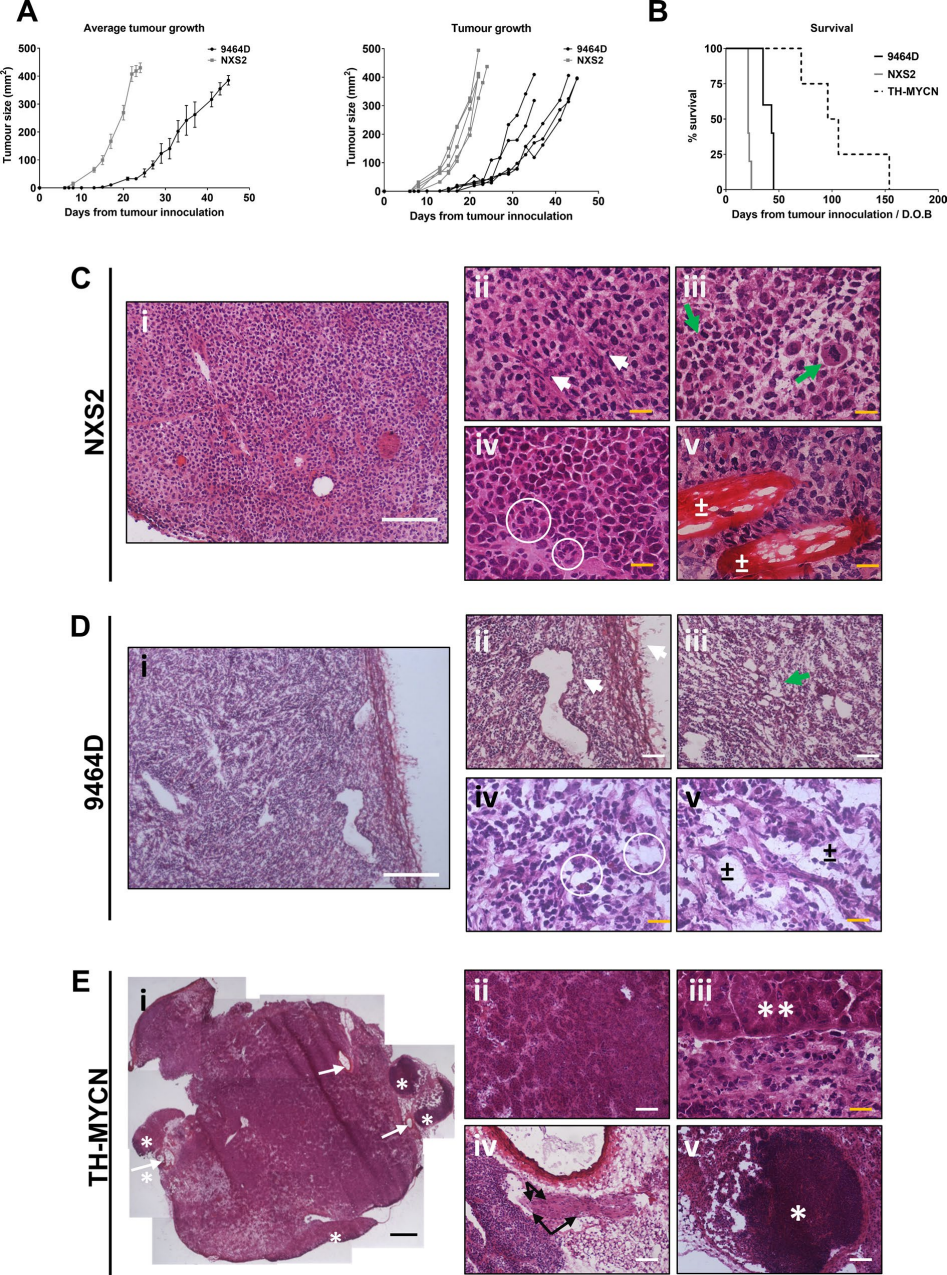
Tumor growth kinetics, survival and histological structure differ between subcutaneous and spontaneous neuroblastoma tumor models. (**A**) AJ or C57BL/6 mice were inoculated subcutaneously with NXS2 or 9464D (respectively) cells. Comparison of tumor growth kinetics of individual mice of NXS2 (grey) and 9464D (black) tumors, and average tumor growth. Tumor size is shown as mm^2^. (**B**) Survival of mice bearing either NXS2 (grey), 9464D (black) or TH-MYCN (dashed) tumors. NXS2 and 9464D survival was measured from date of inoculation. TH-MYCN survival is measure from date of birth. n = 5. (**C**) ex vivo tumors were frozen in OCT and stained by H&E. For NXS2 tumor: (i) homogeneous appearance of the tissue; (ii) collapsed blood vessels; (iii) Mitotic figures; (iv) pseudorosettes; (v) muscle fibers. (**D**) For 9464D tumor: (i) loosely packed appearance with thick capsule; (ii) Large dilated blood vessel and thick capsule around tumor edge; (iii–v) demonstration of ‘holes’ throughout tumor. (**E**) For TH-MYCN tumor: (i) transverse section shows high complexity of the tumor microenvironment with visibly enlarged vessels ( →) and tertiary lymphoid structures surrounding the tumor mass (*); (ii) islands of cancer cells divided by fibrous septa; (iii) tertiary lymphoid structure; (iv) border between tumor cells (bottom) and adrenal gland (top) with ganglion cells in it; (v) cross section of a nerve with embedded ganglion cells, surrounded by cancer cells, fat tissue and enlarged arteriole. Scale bars, 100 µm.

For both subcutaneous models, tumors reliably grew in all mice, over a period of a few weeks. The NXS2 tumors exhibited faster growth kinetics compared to 9464D tumors, with tumors becoming palpable earlier after inoculation and median survival of 21 days and 43 days from tumor inoculation respectively (Fig. 1A,B). All heterozygous TH-MYCN mice developed tumors, with survival ranging from 70 to 160 days after birth (Fig. 1B).

### Tumors in transgenic TH-MYCN mice display complex histological heterogeneity

The histological profile of human neuroblastoma is highly heterogeneous, with varying degrees of neuronal differentiation. Common features include nodular growth pattern with delicate fibrous septae, high vascularization with areas of necrosis, haemorrhage and calcification. Approximately one third of cases contain pseudorosettes around blood vessels or Homer Wright rosettes around neuropil. Undifferentiated tumors may also exhibit pleomorphic, spindle or anaplastic tumor cells. Differentiating tumors consist of differentiating neuroblast cells, and may have Schwannian stromal with mature or maturing ganglion cells^21^. NXS2, 9464D and TH-MYCN were studied macroscopically and histologically, to investigate if they shared similar features to human tumors. Subcutaneous NXS2 tumors showed a blood-filled and fragile texture, with high levels of vascularisation within a capsule, while in comparison, 9464D tumors were pale in appearance, and contained by thick capsule (Supplementary Fig. S1). Transgenic TH-MYCN mice developed large tumor masses in the abdominal cavity, with adhesion to the spine, with one or both kidneys encased in the tumor mass. These tumors had a very compact texture, were heavily vascularised and appeared heterogeneous with areas of necrosis (Supplementary Fig. S1). Histologically, NXS2 tumors consisted of homogeneous tumor cells interspersed by fibrous tissue and blood vessels (Fig. 1Ci). Sections showed collapsed vessels (Fig. 1Cii), mitotic figures (Fig. 1Ciii), formation of pseudorosettes (Fig. 1Civ) and tumor growth between muscle fibre bundles (Fig. 1Cv). 9464D tumors were clearly capsulated, and contained large CD31 + vessels throughout the tumor (Fig. 1Di + ii; Supplementary Fig. S2). The tumors were less dense than NSX2 and punctuated (Fig. 1Diii,iv + v). Some of the empty space within the tumor appears to be microvessels as determined by CD31 and LYVE1 staining (Supplementary Fig. S2), however there is noticeably a lack of blood cells within them. As previously reported^18,22^, TH-MYCN tumors were histological complex (Fig. 1Ei), with islets of multinucleated neuroblastoma cells divided by septae of fibrous tissue (Fig. 1Eii). Apoptotic centres could be observed by chromatin condensation in cell nuclei. Residual adrenal glands with ganglion cells (Fig. 1Eiii) and cross-sections of nerves with embedded ganglion cells (Fig. 1Eiv) were commonly observed. Enlarged and compressed CD31^+^ vasculature could be seen throughout the section, mainly seen in the fibrous septae (Supplementary Fig. S2). Interestingly, in 4 out of 5 specimens, LYVE-1^+^ vessels were filled with neuroblastoma cells (Supplementary Fig. S2), a vascular feature previously found also in samples from patients^23,24^. Finally, tertiary lymphoid structures were found adjacent to the tumor mass in 3 out of 5 specimens (Fig. 1Ev). These areas were positive for CD3 and B220 by immunohistochemistry (Supplementary Fig. S3), confirming their lymphoid lineage. The heterogeneous structure of TH-MYCN tumors with residual tissues from area of origin and vascular anomalies all represent features that recapitulate the heterogeneities found in human tumors.

### Murine neuroblastomas express GD2 and low levels of MHC class I

**Figure 2:**
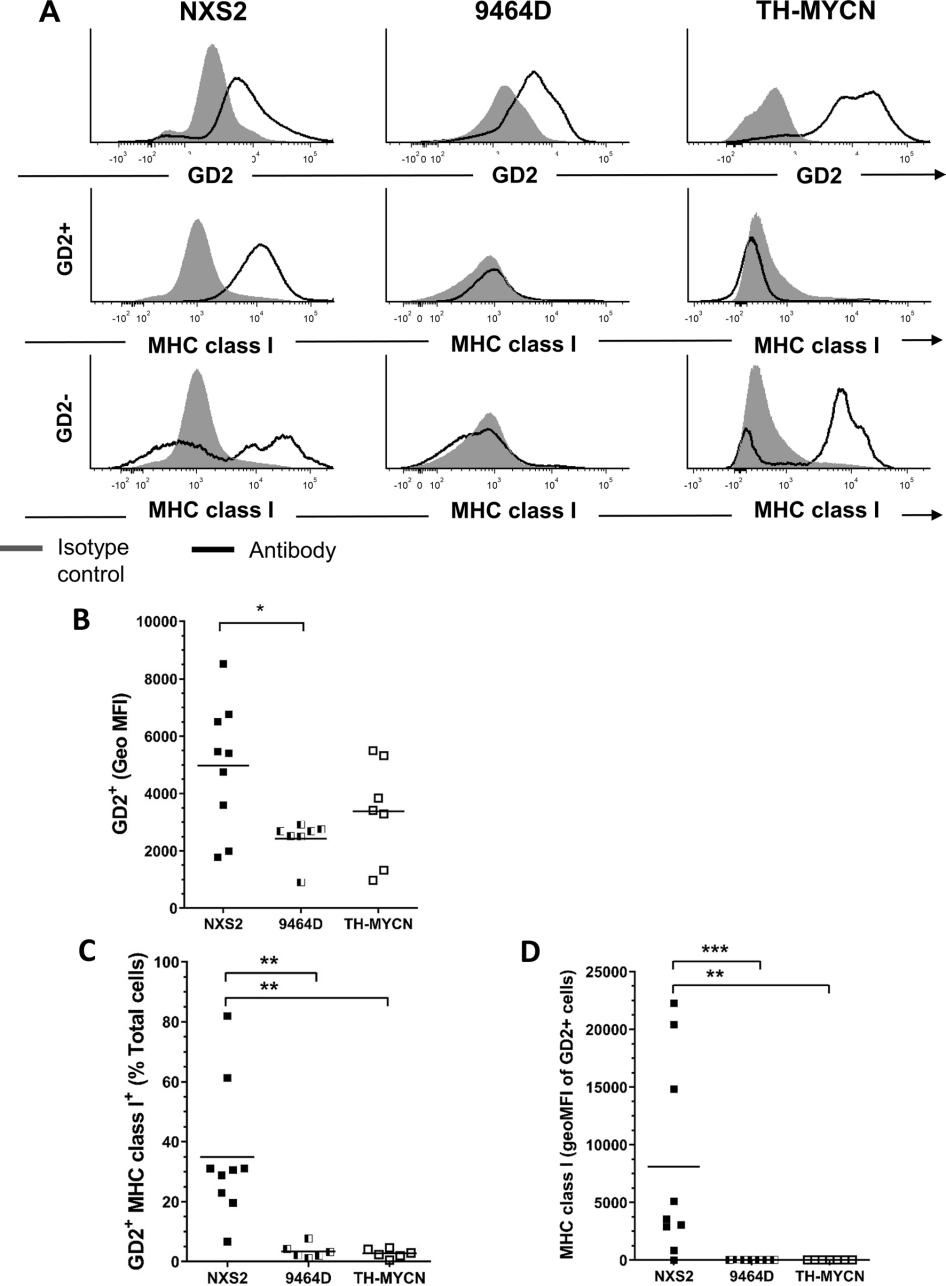
GD2 and MHC Class 1 expression in NXS2, 9464D and TH-MYCN mouse neuroblastoma tumors. (**A**) Ex vivo tumors were disaggregated and stained for flow cytometry analysis. Representative histograms of GD2 and MHC I expression on NXS2, 9464D and TH-MYCN tumors. Expression of MHC I is shown on both GD2+ and non-GD2+ cells. Grey—isotype control; black line—anti-GD2 or MHC I antibody. (**B**) Quantitation of flow cytometry analysis of GD2 expression in ex vivo tumors by geo MFI. (**C**) Percentage of GD2+ MHC I+ cells and (**D**) MHC I expression as geo MFI on GD2+ cells. n = 9 (NXS2), n = 7 (9464D + TH-MYCN). Unpaired t-test, significance was assessed as: **< 0.01, ***< 0.001.

Human neuroblastomas are characterized by high levels of expression of GD2^7,8^ and down regulation of human leukocyte antigen class I (HLA-1)^25^, potentially favouring immune escape^26^. The mouse models were analyzed by flow cytometry (gating shown in Supplementary Fig. S4) to investigate if they shared these properties. GD2 expression was observed on all 3 tumors; expression on NXS2 and TH-MYCN tumors was very variable (Fig. 2B) and 9464D tumors had lower, and more homogenous, levels of expression. In vitro, GD2 expression is documented for NXS2 cells^19^, whereas for 9464D cells there have been conflicting reports of whether they express GD2 in vitro^14,17^. Comparable to human neuroblastoma, GD2^+^ cells in both the 9464D and TH-MYCN models showed significant low level expression of MHC class I, whereas expression was preserved in NXS2 (Fig. 2C,D). In the 9464D model, MHC I downregulation was also observed on both GD2^+^ and GD2^−^ tumor cells (Fig. 2A), but maintained on normal splenocytes (Supplementary Fig. S5).

### Splenic immune cell populations in murine neuroblastoma tumor bearing mice

**Figure 3:**
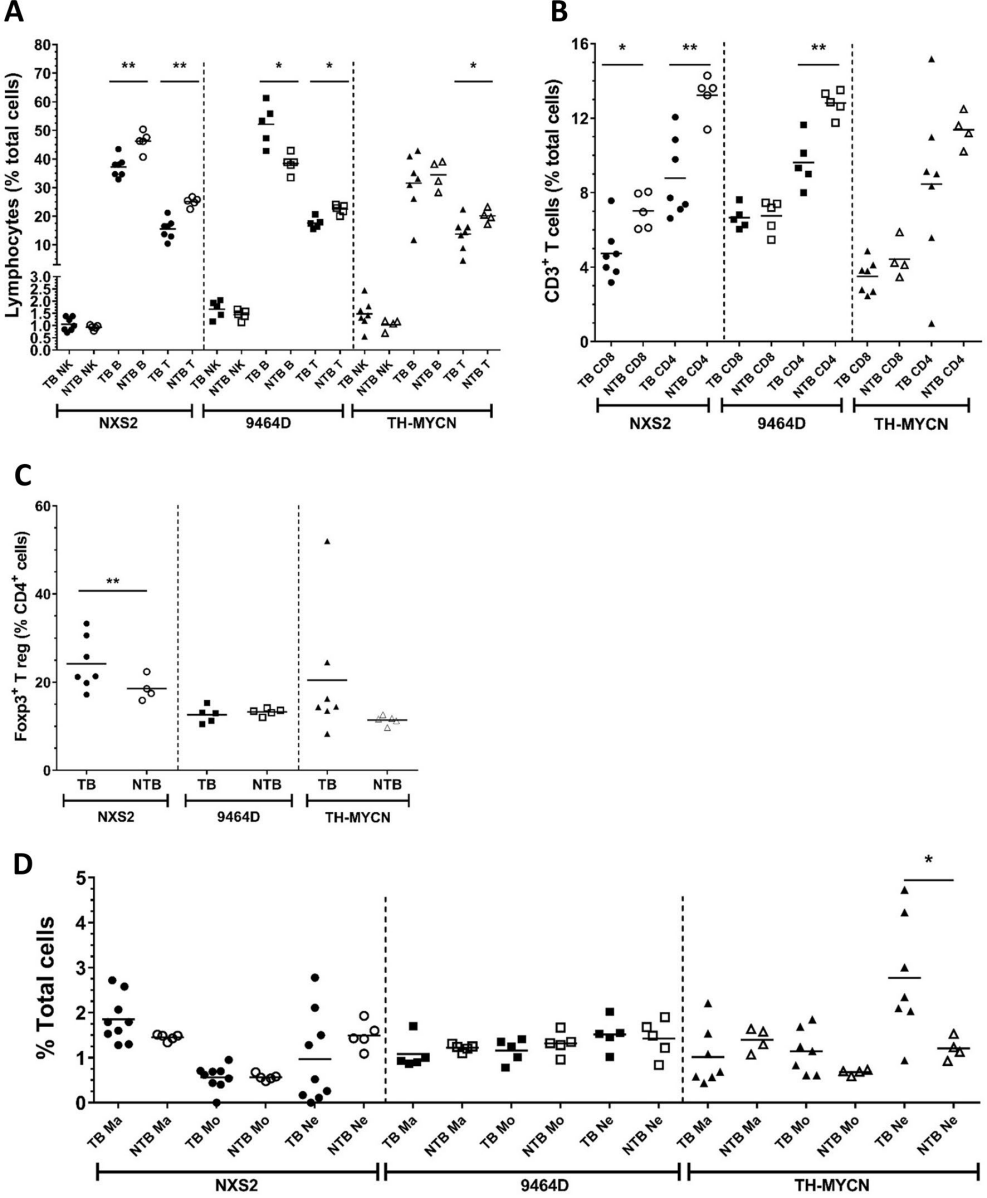
Immunophenotyping by flow cytometry of AJ, C57BL/6 and TH-MYCN tumor or non-tumor bearing spleens. (**A**) Spleens were harvested from either AJ, C57BL/6 or TH-MYCN (tumor bearing or non-tumor bearing) mice and processed into a single suspension for flow cytometry. Gating shown in Supplementary Figs. S4, S6. NK, B and T cells are shown for each strain. (**B**) Proportion of CD8^+^ and CD4^+^ T cells are shown for each strain, with (**C**) demonstrating proportion of Tregs as a percentage of CD4^+^ T cells. (**D**) Percentage of myeloid cells, macrophages, monocytes and neutrophils are demonstrated for each strain. Proportions of cells are shown as a percentage of total cells (singlets) unless otherwise stated. n = 5 per group (AJ and C57BL/6) or 4 per group (TH-MYCN). Significance calculated using Unpaired t-test between TB and NTB mice within each strain, with *< 0.05, **< 0.01, ***< 0.001.

In order to assess if the presence of tumour leads to systemic modulation of the immune system, multi-color flow cytometry was used to quantify lymphocyte populations in the spleen and tumor tissues of the three models (gating demonstrated in Supplementary Figs. S4 and S6). For all three strains, tumor bearing (TB) mice demonstrated a small but significant decrease in the percentage of splenic CD3^+^ T cells, compared to non-tumor bearing (NTB) control mice (Fig. 3A). A significant decrease in CD4^+^ cells was demonstrated in both NXS2 and 9464D TB mice, with variability in CD4^+^ cells seen in spleens of TB TH-MYCN mice (Fig. 3B). NXS2 TB mice also showed a significant decrease in CD8^+^ T cells compared to NTB mice. Treg (CD4^+^ FoxP3^+^) cells were only significantly altered in TB NXS2 mice with a slight increase noted compared to NTB mice was noted (Fig. 3C). B cells were significantly decreased in TB NXS2 mice, but significantly increased in mice bearing 9464D mice, demonstrating tumor specific effects. No significant changes were seen in the proportions of NK cells (Fig. 3A). Finally, myeloid populations within spleens were also assessed in both TB and NTB mice (Fig. 3D). No significant changes were demonstrated in any of the models, apart from a significant increase in splenic neutrophils in TB TH-MYCN mice compared to NTB controls.

### Tumor-infiltrating lymphocyte populations in murine neuroblastoma

**Figure 4:**
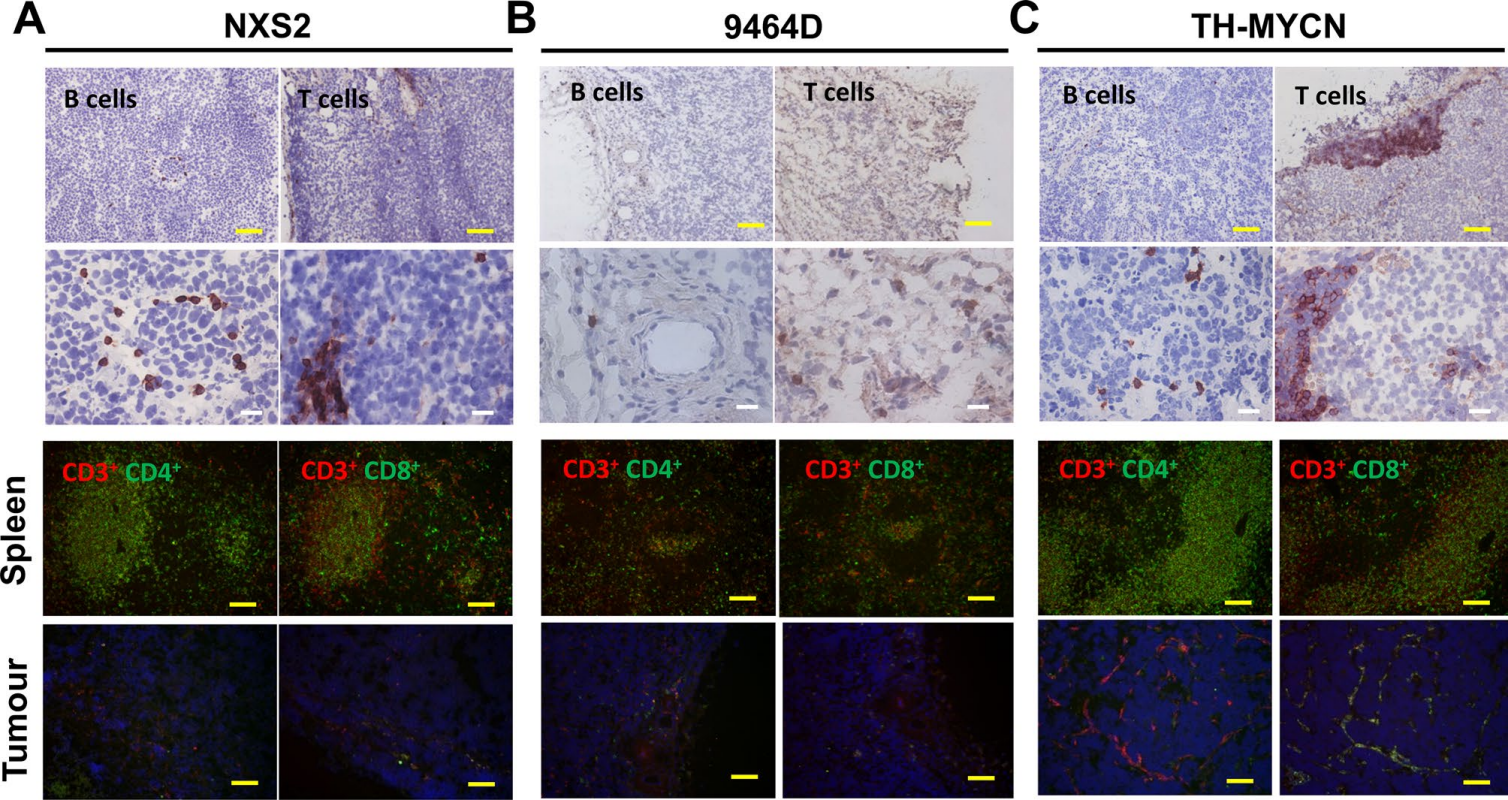
Distribution of TIL populations within NXS2, 9464D and TH-MYCN tumors. (**A**) Ex vivo tumors were frozen in OCT. Either IHC or IF was performed to analyze the distribution and location of T cell subsets and B cells within the tumor mass. B + T cell distribution is demonstrated for NXS2 tumors by IHC staining for B220 (left—B cells) or CD3 (right—T cells). Images are shown at either ×4 magnification (top) or ×10 magnification (bottom). Dual staining of CD3 and CD4 (left) or CD8 (right) by IF of frozen NXS2 spleens (top) or tumors (bottom). Red = CD3, green = CD4/CD8, blue = DAPI. Same is shown in (**B**) for 9464D and (**C**) for TH-MYCN. Representative images are shown. Scale bars, 100 µm.

**Figure 5:**
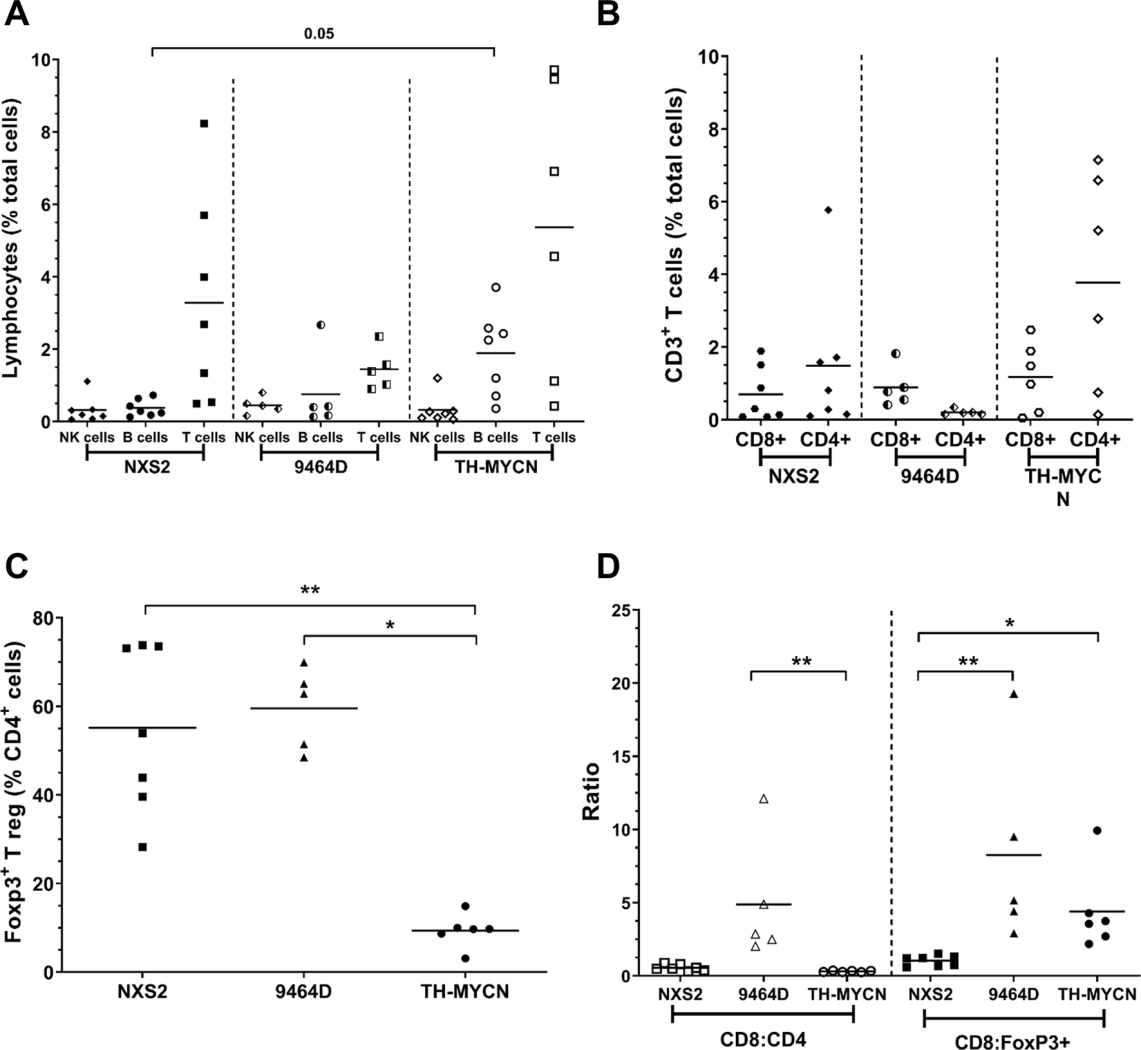
Quantification of TILs within tumors shows variability between NXS2, 9464D and TH-MYCN models. (**A**) Ex vivo tumor and spleens were disaggregated into single cell suspensions for detailed immunophenotyping by flow cytometry as detailed in methods. Gating shown in Supplementary Fig. S6. Proportions of NK, B and T cells are shown, with (**B**) percentages of CD4^+^ and CD8^+^ T cells within tumors. (**C**) Proportion of Treg cells as a percentage of CD4^+^ cells. (**D**) CD8:CD4 ratio (left side) and CD8^+^:FoxP3^+^ ratio (right side) is demonstrated for NXS2, 9464D and TH-MYCN tumors. All proportions are shown as percentage of total cells (singlets) unless otherwise stated. n = 7 (NXS2 and TH-MYCN) and n = 5 (9464D). Significance calculated using Kruskal Wallis test with Dunn’s multiple comparisons, with *< 0.05, **< 0.01, ***< 0.001, ****< 0.0001.

Although human neuroblastoma is considered a poorly immunogenic tumor, activated tumor infiltrating lymphocytes (TILs) have been reported^27^ and their presence appears to correlate favourably with clinical outcome^28^. We investigated TILs in the three murine models by IHC, IF (Fig. 4) and multi-color flow cytometry (Fig. 5), analysing NK cells, B cells and T cell populations. Similar to human neuroblastoma, the numbers of T and B cells are relatively low in all 3 models as demonstrated by both IHC and IF (Fig. 4A–C). In TH-MYCN tumors, most of the CD3^+^ cells are concentrated within tertiary lymphoid structures (Fig. 4C + Supplementary Fig. S3), with most of the lymphocytes in all three models located around the margins, rather than penetrating the tumor mass. To quantitatively assess TILs, flow cytometry was utilised (gating demonstrated in Supplementary Fig. S6). NK cell numbers are similarly low in all the models. B and T cell numbers are highest in TH-MYCN tumors (Fig. 5A). In all 3 models, T cells account for the highest proportion of TILs, however proportions of CD8^+^ and CD4^+^ cells differed, with TH-MYCN and NSX2 tumors having more infiltrating CD4^+^ cells, and 9464D tumors having a predominantly CD8^+^ T cell infiltrate (Fig. 5B).

A significant difference in Treg numbers was seen between tumors (Fig. 5C) with NXS2 and 9464D having a higher percentage (55.14% and 59.55% respectively) of FoxP3 + Treg in the CD4^+^ population, in comparison with TH-MYCN (9.36%). When observing the CD8^+^:CD4^+^ T cell ratio (Fig. 5D), 9464D tumors have a significantly higher ratio (4.89) than TH-MYCN (0.31). Finally, both TH-MYCN and 9464D had similar CD8^+^:FoxP3^+^ ratios within the tumor, which were significantly higher than in NXS2 tumors.

### Macrophage phenotyping and distribution in the neuroblastoma microenvironment

**Figure 6:**
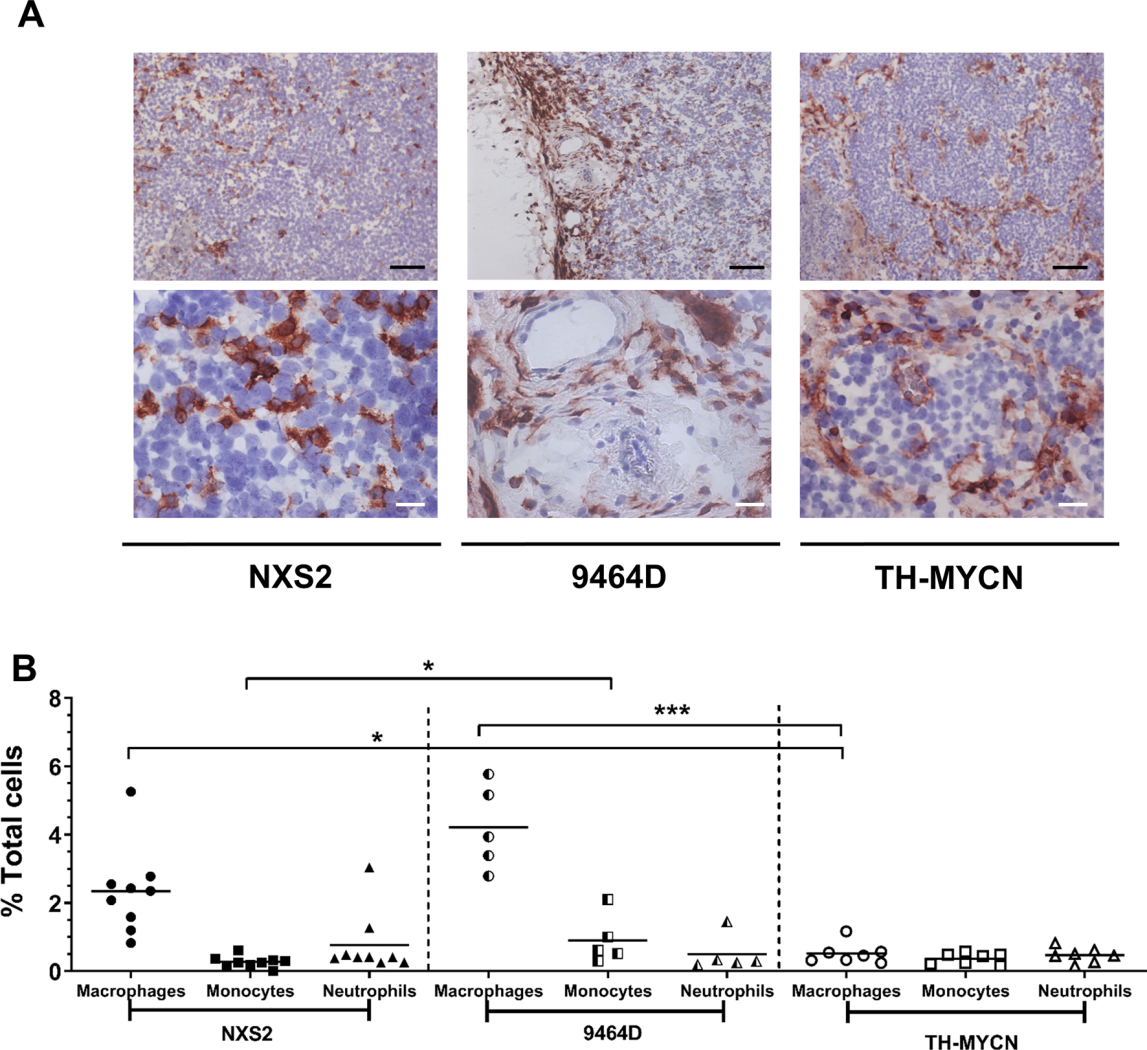
Myeloid cells infiltration in the murine NXS2, 9464D and TH-MYCN neuroblastoma microenvironment. (**A**) Ex vivo tumors were frozen in OCT. IHC for F4/80 expression was performed to analyze macrophages within the tumor mass of (from left to right) NXS2, 9464D and TH-MYCN tumors. Representative images are shown for each tumor type. (**B**) Ex vivo tumor and spleens were disaggregated into single cell suspensions for detailed immunophenotyping by flow cytometry as detailed in methods. Gating shown in Supplementary Fig. S4. Proportions of myeloid subsets in tumors from (left to right) NXS2, 9464D and TH-MYCN tumor bearing mice. All proportions are shown as percentage of total cells (singlets). n = 9 (NXS2), n = 7 (TH-MYCN) and n = 5 (9464D). Significance calculated using Kruskal Wallis test with Dunn’s multiple comparisons, with *< 0.05, **< 0.01, ***< 0.001, ****< 0.0001. Scale bars, 100 µm.

We then assessed the distribution of myeloid cells in the spleen and tumor microenvironment. Figure 6A shows representative images of IHC for the macrophage marker F4/80 for each of the three tumor models. Both subcutaneous models, showed dispersed F4/80^+^ cells across the tumors. However in the 9464D model, there is some evidence of clustering of macrophages within the capsule at the margins. TH-MYCN however, appear to have distinct macrophage localisation to the stromal tissue, where the stroma creates ‘islets’ of tumor cells, similar to human neuroblastoma.

Using flow cytometry, myeloid cell populations were assessed in more detail (gating demonstrated in Supplementary Fig. S4). In the tumor (Fig. 6B), neutrophil infiltration was consistent between all three models. Monocyte percentages were also similar between TH-MYCN and NXS2 models with a significantly higher monocyte infiltration in 9464D tumors, compared to NXS2 (0.91% and 0.28% respectively). Macrophage infiltration was variable between the three models. NXS2 (2.3%) and 9464D (4.21%) were shown to have a significantly higher numbers of macrophages as a percentage of total cells when compared to TH-MYCN tumors (0.52%).

**Figure 7:**
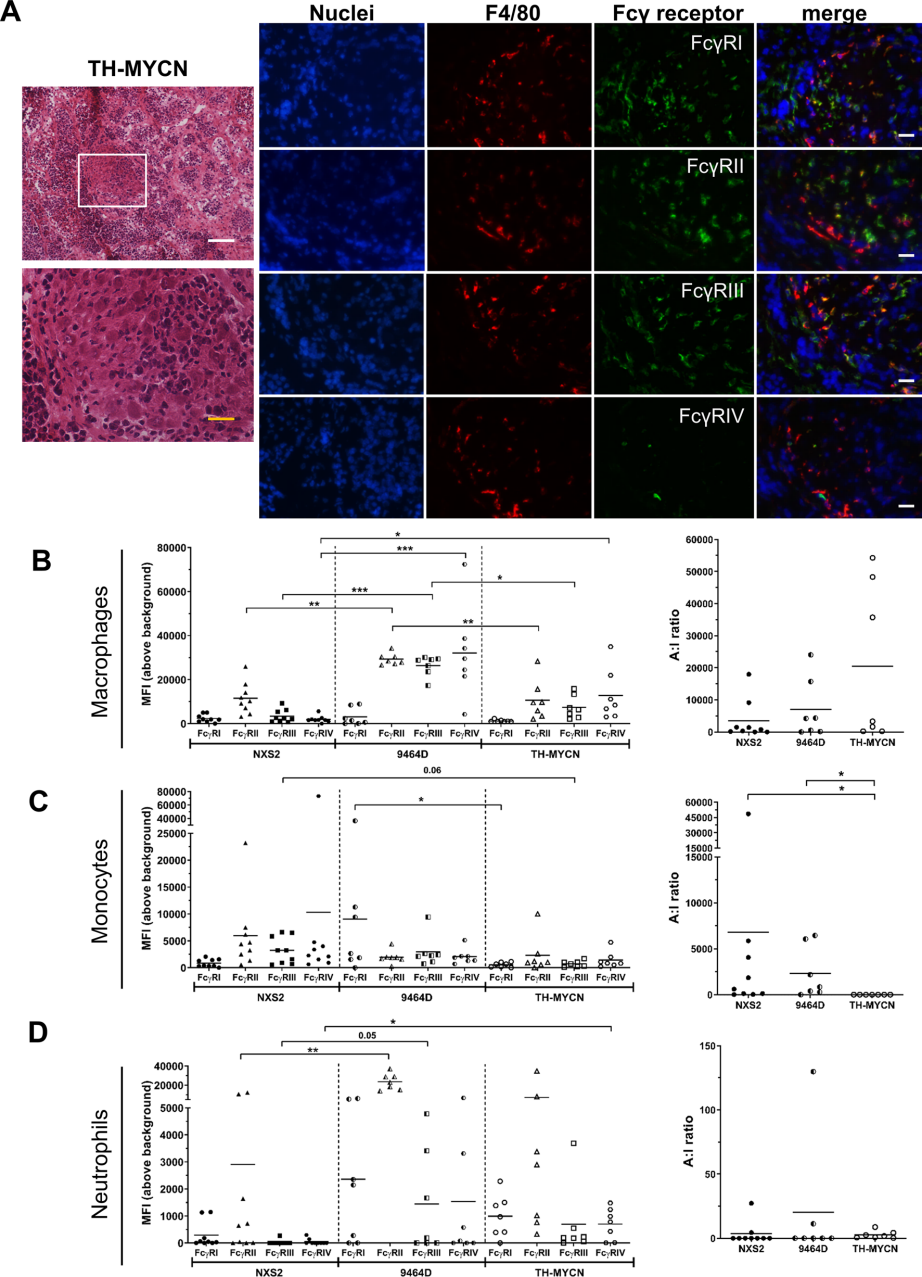
FcγR expression on myeloid cell infiltration in murine neuroblastoma microenvironment. (**A**) TH-MYCN tumors were frozen in OCT. H&E staining and IF for F4/80 and murine FcγRs were performed to demonstrate the distribution and location of macrophages and their receptor expression within the TH-MYCN tumor mass. Blue = DAPI, Red = F4/80, green = (from top to bottom) FcγRI, FcγRII, FcγRIII and FcγRIV. Representative images are shown. (**B**) Tumor and spleens were disaggregated into single cell suspensions for detailed immunophenotyping by flow cytometry as detailed in methods. Gating shown in Sup Fig. 4. FcγR expression was demonstrated as mean fluorescence intensity (MFI) with corresponding activatory:inhibitory (A:I) ratio for macrophages, (**C**) monocytes and (**D**) neutrophils, for (left to right) NXS2, 9464D and TH-MYCN tumors. n = 9 (NXS2) and n = 7 (9464D + TH-MYCN). Significance calculated using Kruskal Wallis test with Dunn’s multiple comparisons, with *< 0.05, **< 0.01, ***< 0.001, ****< 0.0001. Scale bars, 100 µm.

Macrophages have been demonstrated to have distinctive roles during tumor development, progression and response to a range of therapies^29^. Macrophages can be classified, somewhat simplistically, into anti-tumorigenic and pro-tumorigenic subsets, based on phenotype and function. Macrophages classified as ‘M1-like’, are suggested to be classically activated, pro-inflammatory and anti-tumorigenic, and good mediators of effector functions during antibody-based immunotherapy where they have been shown to mediate antibody dependent cellular phagocytosis (ADCP). Alternatively activated ‘M2-like’ macrophages, are suggested to be anti-inflammatory and pro-tumorigenic, and therefore favor tumor progression, angiogenesis and immune escape^30^. The phenotype of tumor-infiltrating myeloid cells and their potential to contribute to mAb dependent effector function can be determined by analysing the expression pattern of activatory (FcγRI, FcγRIII, FcγRIV) and inhibitory (FcγRII) Fcγ receptors^31,32^ and then calculating an A (activatory):I (inhibitory) receptor expression ratio. We investigated the phenotype of the myeloid populations infiltrating the tumor models by comparing the expression levels of their Fcγ receptors (Fig. 7A–D). Interestingly, within the TH-MYCN tumors clusters of F4/80+ cells could be identified. Figure 7A shows an example of the differential expression of the FcγR within F4/80^+^ cells of the cluster, with the majority expressing FcγRI, FcγRII and FcγRIII, with only a few expressing FcγRIV. This highlights the heterogeneous expression of FcγRs within TAMs in this particular tumor model.

Further to this, FcγR expression was analyzed using flow cytometry (gating shown in Supplementary Fig. S4). FcγR expression was seen to be highly variable on TAMs (Fig. 7B). Between NXS2 and TH-MYCN TAMs the only difference seen was a higher expression of FcγRIV. However expression of all FcγRs (except FcγRI) on TAMs in 9464D tumors are significantly different compared to either NXS2 or TH-MYCN. Of note, expression of the inhibitory receptor FcγRII was significantly higher on 9464D TAMs compared to NXS2 and TH-MYCN. However, a heightened expression of activatory receptors FcγRIII and FcγRIV on 9464D compared to NXS2 and TH-MYCN TAMs was also noted. Tumor infiltrating monocytes are relatively consistent between the three models. The only significant difference seen is higher levels of FcγRIII on monocytes in NXS2 tumors compared to TH-MYCN (Fig. 7C). However, TH-MYCN infiltrating monocytes were demonstrated to have significantly lower A:I ratios compared to the other models. Finally, tumor infiltrating neutrophils are shown to have variable FcγR expression between the three models (Fig. 7D). Interestingly, the levels of inhibitory receptor FcγRII was significantly higher on neutrophils infiltrating 9464D tumors when compared to NXS2 infiltrating neutrophils. Expression of the three activatory receptors is highly variable within and between the three models (Fig. 7D).

### Cyclophosphamide creates a therapeutic window for testing monoclonal antibody-based immunotherapy in TH-MYCN mice

**Figure 8:**
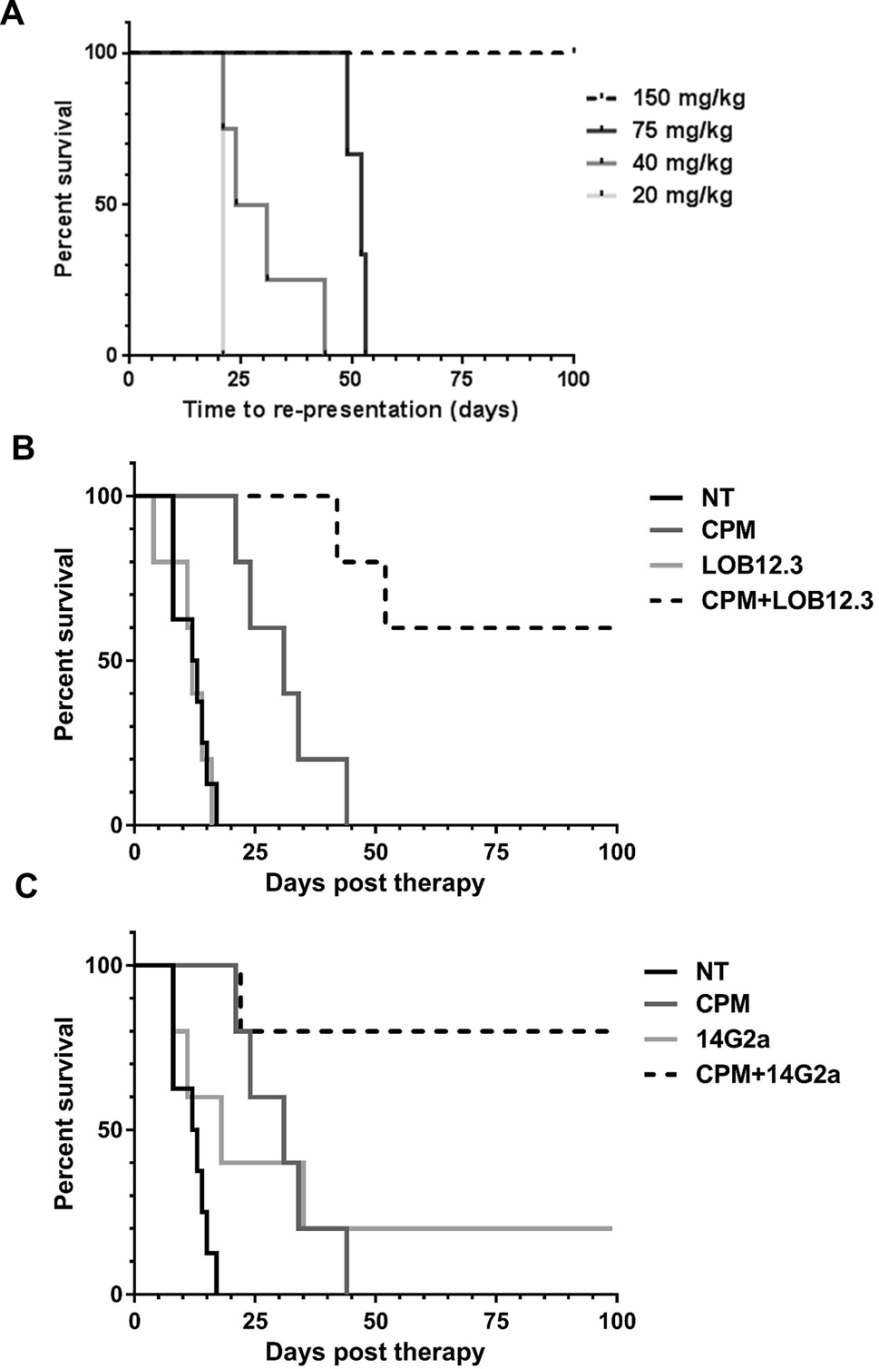
Immunotherapy increases survival of TH-MYCN mice in a MRD setting, compared to immunotherapy alone. (**A**) Tumor-bearing TH-MYCN transgenic mice were treated i.p. with cyclophosphamide (CPM) alone, or 150 µg anti-GD2 (14G2a) mAb alone, or 150 µg anti-4-1BB (LOB12.3) mAb alone, or combinations thereof. Kaplan–Meier curves were generated. Titration of CPM doses to achieve a model of minimal residual disease (MRD). Time taken for tumors to re-present was used as end point. n = 2 (150 mg/kg), n = 3 (75 mg/kg), n = 5 (40 mg/kg) and n = 1 (20 mg/kg). (**B**) Combination therapy of 40 mg/kg CPM with LOB12.3 in TH-MYCN tumor bearing mice. (**C**) Combination therapy of CPM with 14G2a in tumor bearing TH-MYCN mice. Given the spontaneous nature of this model, controls accrue over time, and have been used for comparison to treatment arms in (**B**) and (**C**).

The previous data demonstrated that the TH-MYCN tumor model appeared to best recapitulate human neuroblastoma in terms of histological structure, heterogeneity and T cell infiltrate. Therefore, we sought to utilise this model to allow for pre-clinical assessment of mAb based immunotherapy. In patients, anti-GD2 antibody-based therapies are generally administered once the bulk of neuroblastoma has been eradicated, in a minimal residual disease (MRD) setting, in order to maintain disease remission and reduce the risk of relapse^9,10^. In addition, recent trials have shown very promising results when anti-GD2 has been given upfront, in conjunction with induction chemotherapy^33^. Furthermore, TH-MYCN tumors have relatively rapid growth kinetics, with a short window of opportunity to treat before mice reach their humane end-point, potentially not allowing enough time for effective immunotherapy. Therefore, we aimed to find identify a dose of chemotherapy that would be sufficient to reduce tumor burden without achieving long term cure, thus creating a therapeutic window during which the effects of immunotherapy could be assessed. As tumors became palpable, TH-MYCN were treated with a single dose of 20, 40, 75 or 150 mg/kg of cyclophosphamide (CPM). Mice injected with 150 mg/kg survived in excess of 100 days. Further groups of mice which developed tumors were treated with a reduced dose of CPM of either 75 mg/kg with a median survival of 52 days, 40 mg/kg (27.5 days) or 20 mg/kg (21 days) (Fig. 8A). 40 mg/kg was selected as a ‘sub-curative’ dose as it gave a mean survival of one month prior to re-presentation with all mice subsequently reaching their humane end-point (< day 50–data not shown). Using this model, we were able to assess the efficacy of tumor targeting anti-GD2 (14G2a) and immune stimulatory anti-4-1BB (LOB 12.3) mAbs in prolonging the survival of TH-MYCN mice and establish proof of principle for this combinatorial approach in a MRD setting. Each antibody was tested alone and in combination with CPM. The combination of CPM + LOB12.3 was more effective than either agent when administered alone, with 60% of mice treated surviving 100 days without re-presentation of tumor. Additionally, mice treated with CPM alone survived significantly longer than mice treated with LOB12.3 alone, although 100% of mice in each group eventually re-presented with tumor (Fig. 8B). The combination of CPM + 14G2a was more effective than either agent alone, with 80% of mice surviving 100 days without re-presentation of tumor. However, there was no significant difference in survival between mice treated with CPM alone and those treated with 14G2a alone, with 100% and 80% of mice re-presenting with tumor respectively (Fig. 8C). In summary, using the TH-MYCN mouse model in a MRD setting, two mAb therapies, with different mechanisms of action, markedly increased survival when compared to monotherapies alone.

## Discussion

Current treatment for neuroblastoma is intensive and associated with significant toxicity. The introduction of anti-GD2 immunotherapy to high-risk neuroblastoma treatment protocols has achieved some improvement in outcomes, but the number of other immunotherapies that have entered clinical trial in this disease is limited. New immunotherapies being investigated in neuroblastoma include checkpoint blockade^34^, T cell therapy^35^ and NKT cell therapies^36^. Neuroblastoma is rare, with approximately 100 cases per year in the UK and therefore, in contrast to adult oncology, the number of clinical trials that can be conducted to assess new therapeutics is limited. It is therefore imperative to maximize the pre-clinical evaluation of new therapeutics, to ensure new agents are appropriately prioritized, and that dosing, scheduling and combination strategies are optimized as much as possible before clinical trial. Although murine models are not an alternative to conducting clinical trials, there is a need for informative tools that enable mechanisms to be established and that help prioritize therapeutics and combinations brought to the clinic for high-risk neuroblastoma. However, pre-clinical evaluation is only possible if appropriate models are available, which recapitulate the biological features of neuroblastoma, within the context of a functioning immune system.

There is relatively sparse data describing the immune environment of human neuroblastoma, but the reported data suggests that tumors downregulate MHC class I, have low T cell infiltrate and are relatively macrophage rich. Furthermore, when T cells are present in tumors, this correlated with outcome^5,27,37–39^.

Here, we compared three murine models of neuroblastoma. Two subcutaneous models (NXS2 and 9464D) and one spontaneous transgenic model (TH-MYCN) were assessed for their resemblance to human disease using a combination of histological techniques and detailed immunophenotyping by flow cytometry of TILs and myeloid cells.

Histology of the three models differed widely, from the homogenous NXS2 tumors, to the complex heterogeneous TH-MYCN tumors. Pseudorosettes and tumor ‘islands’ separated by septae are characteristic of human neuroblastoma^21^ and although NXS2 and 9464D showed some evidence of these structures, (Fig. 1C,D), these features were more common in the TH-MYCN tumors (Fig. 1E), which were found to have a very similar histological structure to human neuroblastoma. Furthermore, these tumors also demonstrated tertiary lymphoid structures, resembling those found in human neuroblastoma^5^. Overall, histologically the TH-MYCN tumors were much more comparable to human neuroblastoma compared to the subcutaneously models.

MHC I downregulation is well documented in human neuroblastoma as a mechanism of immune escape^25,26,40–43^. Both the TH-MYCN and 9464D models show downregulation of MHC I on GD2^+^ cells within tumors (Fig. 2A–C). NSX2 tumor cells maintained expression of MHC Class I, suggesting this model may be less useful, particularly for the assessment of T cell mediated immunotherapies, where a Class I expressing model may over-estimate potential efficacy. All three models expressed GD2, in keeping with human neuroblastoma^8,44–46^, however, expression was low in 9464D tumors, as previously reported^17^. This low GD2 expression can be increased transduction of GD2 and GD3 synthases^17^. Modelling comparative GD2 expression levels are important, given that a large proportion of potential immunotherapies for neuroblastoma target GD2.

Comparison of splenic immune populations from tumor bearing (TB) and non-tumor bearing (NTB) mice of the same strains as the tumor models demonstrated tumor induced systemic effects. Presence of tumor in all three models lead to a reduction in CD4^+^ T cells, with a greater proportion of these cells being FoxP3^+^ Treg cells within the spleens of mice bearing NXS2 tumors (Fig. 3C). Furthermore, in TH-MYCN TB mice a greater percentage of neutrophils was demonstrated compared to the NTB mice. The increase in Tregs and neutrophils in the spleen of TB mice of these models suggest that the tumors are having systemic inflammatory and immunoregulatory effects.

It is important that the preclinical models used to test potential immunotherapies exhibit similar immune cell infiltrates in their tumor microenvironments. It has previously been reported that lymphocytic infiltration has been observed in human neuroblastoma tumors^5, 27,37,38^, however the number of infiltrating lymphocytes were low. Using a combination of IHC, IF and flow cytometry we assessed the immune infiltrates in NXS2, 9464D and TH-MYCN tumors as shown in Figs. 3, 4, 5, 6, 7. In human neuroblastoma, B cells and NK cells have been reported as absent or rare within the tumor mass^5^, which, was also demonstrated in the murine models, apart from high levels of B cells in TH-MYCN tumors (Fig. 5A). Coughlin et al., demonstrated using flow cytometry that human neuroblastoma samples had low levels of infiltrating CD3^+^ T cells, at < 5% of all cells^5^. Another study by Carlson et al., looked at 8 primary neuroblastoma samples and found the presence of CD3^+^ T cells which varied considerably between samples, with a mean of 4.5%^27^. We have demonstrated here that all three murine models have a low CD3^+^ infiltrate, with lowest numbers observed in 9464D tumors (Fig. 5A). Furthermore, it has previously been demonstrated that many of the CD3^+^ T cells and lymphocytes which infiltrate human tumors are located in the periphery around the margins of tumors^5,38^. Here we have shown similar findings with all three murine models, with the majority of CD3^+^ cells being present around the edges of tumors (Fig. 4). When comparing the ratio of T cell subsets, 9464D tumors have more CD8^+^ than CD4^+^ T cells (Fig. 5B). This has also been reported in human tumors, which were found to have a CD4:CD8 ratio of 0.82^27^. Importantly, both tumors of subcutaneous origins (NXS2 and 9464D), demonstrated significantly higher levels of Treg cells compared to the spontaneously developing TH-MYCN tumors (Fig. 5C). This may be due to a more inflammatory environment in the subcutaneous tumors as the cells have been injected into this site and grow rapidly, rather than appeared spontaneously over a period of time as in TH-MYCN tumors. It has been reported previously that human neuroblastoma tumors have a fairly substantial FoxP3^+^ cell infiltration of between 40 and 55% of CD4^+^ cells, however this was only demonstrated in a small cohort of 3 patients^27^. The number of Tregs seen in NXS2 and 9464D is high at around 60% of CD4^+^ cells compared to TH-MYCN, which have a Treg infiltrate of around 10% CD4^+^ cells. Tregs play an important role in controlling the immune response to tumors and several current immunotherapies target these cells to modulate this. However, given the very limited data as to the Treg infiltration in human tumors, it is difficult to assess which of the pre-clinical models best recapitulates this.

It has been previously reported that CD68^+^ and CD163^+^ TAMs are present in human neuroblastoma samples^39,47–49^. We found macrophages present in all three murine tumors (Fig. 6). Interestingly however, we demonstrated that both NXS2 and 9464D had a higher percentage of infiltrating macrophages compared to the spontaneous TH-MYCN tumors (Fig. 6B). As mentioned previously, this most likely reflects a more inflammatory microenvironment in the subcutaneous tumors. Despite the lack of comparable data but given their importance to mAb effector function, the proportion, location and expression of FcγR was analyzed on myeloid cells within the tumor microenvironments of the three murine models (Figs. 6, 7). Although no data on FcγR expression on human neuroblastoma samples has yet been published, it is important to assess their expression when considering the use of immunotherapy in the form of antibody therapy. This is due to various isotypes of mAb interacting with different FcγRs and engaging with a wide range of affinities, which has in turn been shown to impact mAb therapeutic efficacy^50^. For our murine models, expression of the 4 murine FcγRs (FcγRI, FcγRII, FcγRIII and FcγRIV) were analyzed (Fig. 7). Expression of the receptors varied greatly between the three models and the different cell types. Importantly, 9464D had high levels of the inhibitory receptor FcγRII on both macrophages and neutrophils, along with higher expression of FcγRIII and IV compared to both NXS2 and TH-MYCN models and a higher A:I ratio on monocytes compared to TH-MYCN tumor monocytes. This may represent a more pro-inflammatory tumor microenvironment within the 9464D model compared to the other two models.

Here we have restricted our assessment to the immune microenvironment in subcutaneous tumors compared to a spontaneous transgenic model. However, this presents the caveat that when comparing subcutaneous xenografts versus endogenous abdominal tumors, the anatomical location and intrinsic tumor biology are inherently different. Therefore, differences within the tumor infiltrates demonstrated here, may in part be due to this disparity between the models. For example, the increased percentage of macrophages found in the subcutaneous models could, at least in part, reflect a more inflammatory environment due to the ectopic injection of tumor cells. It is noteworthy that orthotopic models using the 9464D cell line have been established. Kroesen et al., have demonstrated previously that the orthotopic implantation of 9464D cell recapitulated human NB more so than subcutaneous tumors^13,14^. Despite this, subcutaneous models are still widely used in cancer research including NB, therefore it is important to know how these models directly compare to each other, and to more translational models, as demonstrated here and detailed above. Subcutaneous models are amenable to setting up large cohorts of mice to enable a wide panel of prospective treatments to be directly compared, this is much more difficult to achieve in more complex orthotopic models which are more resource intensive, requiring specific surgical expertise, and means monitor tumor growth using imaging, such as via the insertion of fluorescent or luminescent reporters, or via CT/MRI scanning. Indeed the presence of fluorescent or luminescent reporters (such as GFP or luciferase respectively), could make the tumor cell lines more immunogenic, and could in themselves alter the immune infiltrates^51^. However, Kroesen et al., demonstrated that this may not be the case in their 9464D orthotopic model^13^.

Data presented here suggests that of the three models, the TH-MYCN spontaneous transgenic mouse may represent human neuroblastomas well due to their histological structure, similar MHC class-I expression on tumor cells, and comparable tumor immune infiltrates such as low levels of CD3^+^ cells. However, as tumors are detected by palpation, they can frequently reach large tumor sizes within 1–2 weeks. This is generally not enough time to allow immunotherapeutics, such as mAbs, to be administered and be effective. Implementation of CT scanning to detect TH-MYCN tumors in mice before they can be palpated could mean earlier detection and therefore a larger window to assess treatments. Furthermore, as TH-MYCN tumors are driven by MYCN amplification it is worth noting that this model may not be appropriate for investigating the biology of MYCN non-amplified high risk neuroblastoma. In addition, given the response exhibited to low dose single agent cyclophosphamide, it may be more sensitive to chemotherapy than human neuroblastoma. Nevertheless, we demonstrate that with established tumors, low, sub-curative doses of cyclophosphamide (40 mg/kg) can be used to reduce tumor burden and generate a therapeutic window to combine with various immunotherapeutics (Fig. 8A). Using direct targeting (anti-GD2) or immunostimulatory (anti-4-1BB) mAbs in a MRD setting was shown to increase survival significantly of tumor bearing TH-MYCN mice compared to single agents alone (Fig. 8B,C). However, it is worth noting that CPM is not used a single agent therapy for treatment of high-risk NB, but data presented here demonstrate a proof-of-concept that the TH-MYCN models can be used to model combination immunotherapy. Furthermore, it has been demonstrated that the metabolism of CPM into its active metabolites in vivo is inherently different between mice and humans^52^. Due to these metabolic differences, mice are regarded as being more sensitive than humans to CPM, therefore the dosing schedule presented here, does not directly translate into patients. Overall, these data suggest that TH-MYCN mice can be used as an effective model to assess immunotherapeutics and combinational therapy.

Here, we present data comparing the most widely used immunocompetent murine models and their ability to model human NB disease. However, xenograph models, using human tissue or cell lines are another model which can be utilized^15,36,53–56^. Until recently these models mainly used immunompromised mice and therefore it would be difficult to study tumor immunology and immunotherapeutics. Recent advancements have shown promising results utilizing NOD *scid* gamma mice reconstituted with human peripheral blood lymphocytes and injected orthotopically with either patient derived or cell line human neuroblastoma cells, permitting the study of human immune cell interaction within a human tumor microenvironment^57^. These sophisticated models are still in their relative infancy with many caveats attached to their use, such as poor reconstitution of certain immune cell compartments, differing MHC between stem cell donor and tumor graft and development of graft versus host disease^58,59^, but they are potentially very promising models for future evaluation and comparison with established murine models.

In summary, due to the spontaneity and location of TH-MYCN tumor development, together with its histology, immune infiltrates and MHC class I expression we believe this model currently best represents the immune environment of NB, although this model does fail to recapitulate bone metastasis and complex heterogeneity as seen in human MYCN amplified neuroblastoma^15,54^. However, pre-clinical testing in this model requires maintenance of a large colony of transgenic mice. In addition, imaging (e.g. ultrasound or CT) may be necessary to detect abdominal tumors at a sufficiently early stage to allow treatment. The 9464D subcutaneous tumor model may be more practical and offer a similar immune microenvironment. Importantly, the 9464D model has been demonstrated to be ‘immunologically cold’ compared to NXS2, and is therefore more representative of human NB^17,60,61^. Although orthotopic models, provide a more representative tumor microenviroment^13^, they have practical limitations compared to subcutaneous models. In this context, the 9464D subcutaneous model permits the testing and optimisation of a large panel of therapies before assessing the most promising in the TH-MYCN model.

## Materials and methods

### Mice

129/svj.1, AJ and C57BL/6 mice were bred and maintained locally. Transgenic TH-MYCN mice were kindly provided by Professor William Weiss, NIH; mice were bred and genotyped locally and heterozygous mice used experimentally^18^. All procedures were carried out with local ethical approval and performed in accordance to the Animals (Scientific Procedures) Act 1986 as set out in project licenses: RRF30/2964 and PB24EEE31.

### Tumor cell lines

GD2-expressing NXS2 cell line (provided by Prof. Holger Lode, Medical University of Greifswald,) were cultured in Dulbecco’s Modified Eagle medium (DMEM, Gibco, Life Technologies), supplemented with 10% heat-inactivated fetal calf serum (Sigma-Aldrich), 2 mM l-Glutamine, 0.1 mM Minimum Essential Medium non-essential amino acids (Gibco, Life Technologies) at 37 °C and 5% CO_2_. Cells were harvested via trypsin–EDTA once 80% confluence was reached. 9464D cells (provided by Dr. Rimas Orentas, NIH) were cultured in RPMI 1640 supplemented with 10% FCS, 2 mM l-Glutamine, 0.1 mM Minimum Essential Medium non-essential amino acids, and 0.36% 2-Mercaptoethanol (Sigma Aldrich) at 37 °C and 5% CO_2_. Cells were harvested at 80% confluency using TripLE express (Gibco, Life Technologies).

### In vivo tumor models

Groups of AJ and C57BL/6 mice were injected subcutaneously with 2 × 10^6^ NSX2 or 5 × 10^5^ 9464D cells respectively. Tumors were measured regularly with calipers and harvested either as described in figure legends, or when set humane endpoints were reached. Heterozygous TH-MYCN mice^18^ were assessed twice weekly for presence of abdominal tumor masses by a trained technician. Upon presentation with palpable tumor, mice were weighed and treated i.p. with 40 mg/kg of cyclophosphamide (CPM) (Sigma-Aldrich) in 200 µL PBS. Mice received either CPM alone, mAb alone, or a combination of both. When treated with mAb, mice were injected i.p. with 150 µg of anti-GD2 mAb (14G2a), or anti-4-1BB (LOB12.3) mAb in 200 µL PBS. Mice were palpated regularly for signs of tumor progression, regression or re-presentation and culled once humane end-point (> ~ 1 cm tumor) was reached. The differing numbers of mice treated within the groups is due to the spontaneity of tumour development in the TH-MYCN heterozygous model.

### Tumor and spleen dissociation

For NXS2 and 9464D models, once tumors reached 10 × 10 mm, tumor and spleen tissue were harvested with half being used for flow cytometry and half frozen in OCT (CellPath) for histological analysis. The same was conducted for TH-MYCN once tumors had reached endpoint. For flow cytometry, both tissues were mechanically disassociated, processed with mouse Tumor Dissociation Kit (Miltenyi Biotech) according to the manufacturer’s instructions. Dissociated cells were passed through a 100 µm cell strainer (Falcon, Corning), spun and resuspended in PBS to obtain a single cell suspension.

### Flow cytometry

Samples were labelled with LIVE/DEAD^®^ Fixable Aqua Dead Cell Stain (Invitrogen, Life Technologies) according to the manufacturer’s instructions. 1 × 10^6^ cells in 100 µL of PBS were directly labelled with antibody panels as stated in “Antibody” section below, 30 min on ice. Cells were then fixed with Red Cell Lysis Buffer (BUF04C, BioRad) and washed with FACS wash buffer containing 1% bovine serum albumin fraction V and 20 mM sodium azide in PBS. For intracellular FoxP3 staining, cells were stained using a FoxP3 Staining Buffer Set (eBioscience) according to the manufacturer’s instructions. Cells were analyzed using a FACS Canto II (BD Biosciences) with data analyzed with either FACS Diva software (BD Biosciences) or FCS Express V3 (De Novo Software).

### Antibodies

All antibodies used for flow cytometry, immunohistochemistry and immunofluorescence are detailed in Supplementary Table S1. For in vivo treatment: the anti-GD2 monoclonal antibody 14G2a was produced in house by secreting hybridoma HB9118 (kindly provided by Prof. Holger Lode, University of Greifswald); the anti-4-1BB (LOB12.3) Rat IgG1^62^ monoclonal antibody was produced in house by secreting hybridoma cell lines and purified from supernatant using Protein A columns (GE Healthcare).

### H&E staining, immunohistochemistry, immunofluorescence

Excised tissue was embedded in OCT (CellPath) and frozen using isopentane on dry ice. 8 µm sections were fixed in acetone and subjected to H&E staining or antibody labelling. For antibody labelling, sections were incubated sequentially with primary antibodies (Supplementary Table S1), followed by: *Immunohistochemistry,* ImmPRESS anti-rat or anti-rabbit IgG peroxidase polymer, Vector NovaRED peroxidase substrate, counterstained with haematoxylin and mounted using Histomount (all reagents, Vector Labs). *Immunofluorescence*, AlexaFluor488-conjugated anti-goat IgG and AlexaFluor568-conjugated anti-rat IgG (Life Technologies), counterstained with DAPI (Molecular Probes) and mounted with Vectashield (Vector Labs). Images were collected using a CKX41 inverted microscope reflected fluorescence system equipped with a CC12 color camera running under Cell B software (Olympus, UK). Composite immunofluorescence images were prepared using Adobe Photoshop (CS6).

### Statistical analysis

Graphs were produced and statistical analyzes performed using a combination of GraphPad Prism (GraphPad) and Excel 2013 (Microsoft Corporation). Either un-paired t-test or Kruskal Wallis test with Dunn’s multiple comparisons were used to compare groups, as stated in figure legends. Kaplan–Meier survival curves were compared by Log-rank (Mantel–Cox) test. Differences were considered significant when *P-*value < 0.05 (*p < 0.05, **p < 0.01, ***p < 0.001, ****p < 0.0001).

### Ethics approval

All animal experiments had ethical approval from the University of Southampton Animal welfare and ethics review board (AWERB).

## Supplementary information

Supplementary Information.

## Data Availability

Raw data used and analyzed during the current study are available from the corresponding author on reasonable request.
