# Supplementary material for: Immune characterization of pre-clinical murine models of neuroblastoma
Source: Sci Rep. 2020 Oct 7;10:16695. doi: 10.1038/s41598-020-73695-9 (PMC7541480; doi:10.1038/s41598-020-73695-9)
Supplement: Supplementary file 1 — Supplementary Information. [file 41598_2020_73695_MOESM1_ESM.pptx]

## Slide 1
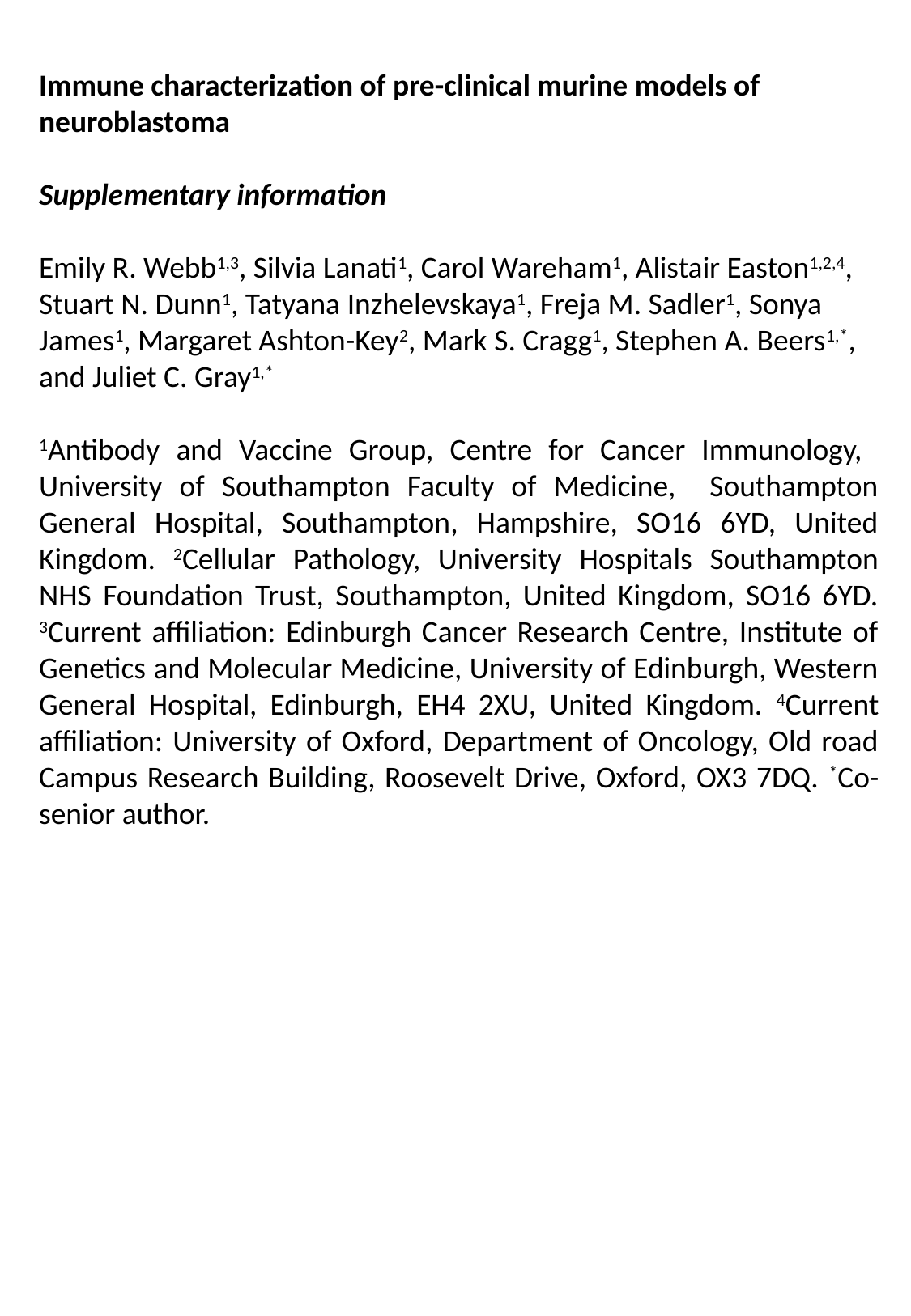

Immune characterization of pre-clinical murine models of neuroblastoma
Supplementary information
Emily R. Webb1,3, Silvia Lanati1, Carol Wareham1, Alistair Easton1,2,4, Stuart N. Dunn1, Tatyana Inzhelevskaya1, Freja M. Sadler1, Sonya James1, Margaret Ashton-Key2, Mark S. Cragg1, Stephen A. Beers1,*, and Juliet C. Gray1,*
1Antibody and Vaccine Group, Centre for Cancer Immunology, University of Southampton Faculty of Medicine, Southampton General Hospital, Southampton, Hampshire, SO16 6YD, United Kingdom. 2Cellular Pathology, University Hospitals Southampton NHS Foundation Trust, Southampton, United Kingdom, SO16 6YD. 3Current affiliation: Edinburgh Cancer Research Centre, Institute of Genetics and Molecular Medicine, University of Edinburgh, Western General Hospital, Edinburgh, EH4 2XU, United Kingdom. 4Current affiliation: University of Oxford, Department of Oncology, Old road Campus Research Building, Roosevelt Drive, Oxford, OX3 7DQ. *Co-senior author.

## Slide 2
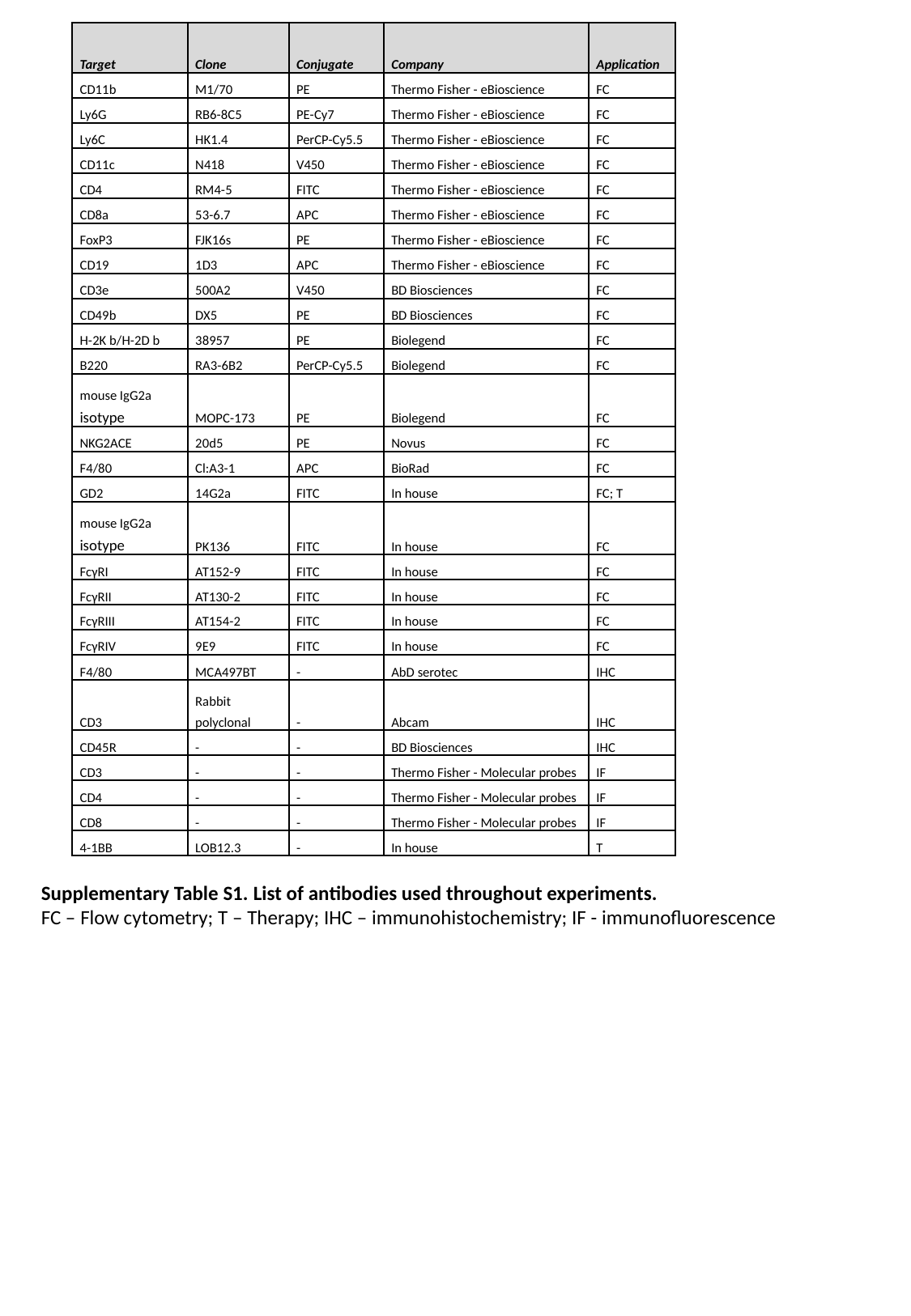

| Target | Clone | Conjugate | Company | Application |
| --- | --- | --- | --- | --- |
| CD11b | M1/70 | PE | Thermo Fisher - eBioscience | FC |
| Ly6G | RB6-8C5 | PE-Cy7 | Thermo Fisher - eBioscience | FC |
| Ly6C | HK1.4 | PerCP-Cy5.5 | Thermo Fisher - eBioscience | FC |
| CD11c | N418 | V450 | Thermo Fisher - eBioscience | FC |
| CD4 | RM4-5 | FITC | Thermo Fisher - eBioscience | FC |
| CD8a | 53-6.7 | APC | Thermo Fisher - eBioscience | FC |
| FoxP3 | FJK16s | PE | Thermo Fisher - eBioscience | FC |
| CD19 | 1D3 | APC | Thermo Fisher - eBioscience | FC |
| CD3e | 500A2 | V450 | BD Biosciences | FC |
| CD49b | DX5 | PE | BD Biosciences | FC |
| H-2K b/H-2D b | 38957 | PE | Biolegend | FC |
| B220 | RA3-6B2 | PerCP-Cy5.5 | Biolegend | FC |
| mouse IgG2a isotype | MOPC-173 | PE | Biolegend | FC |
| NKG2ACE | 20d5 | PE | Novus | FC |
| F4/80 | Cl:A3-1 | APC | BioRad | FC |
| GD2 | 14G2a | FITC | In house | FC; T |
| mouse IgG2a isotype | PK136 | FITC | In house | FC |
| FcγRI | AT152-9 | FITC | In house | FC |
| FcγRII | AT130-2 | FITC | In house | FC |
| FcγRIII | AT154-2 | FITC | In house | FC |
| FcγRIV | 9E9 | FITC | In house | FC |
| F4/80 | MCA497BT | - | AbD serotec | IHC |
| CD3 | Rabbit polyclonal | - | Abcam | IHC |
| CD45R | - | - | BD Biosciences | IHC |
| CD3 | - | - | Thermo Fisher - Molecular probes | IF |
| CD4 | - | - | Thermo Fisher - Molecular probes | IF |
| CD8 | - | - | Thermo Fisher - Molecular probes | IF |
| 4-1BB | LOB12.3 | - | In house | T |
Supplementary Table S1. List of antibodies used throughout experiments.
FC – Flow cytometry; T – Therapy; IHC – immunohistochemistry; IF - immunofluorescence

## Slide 3
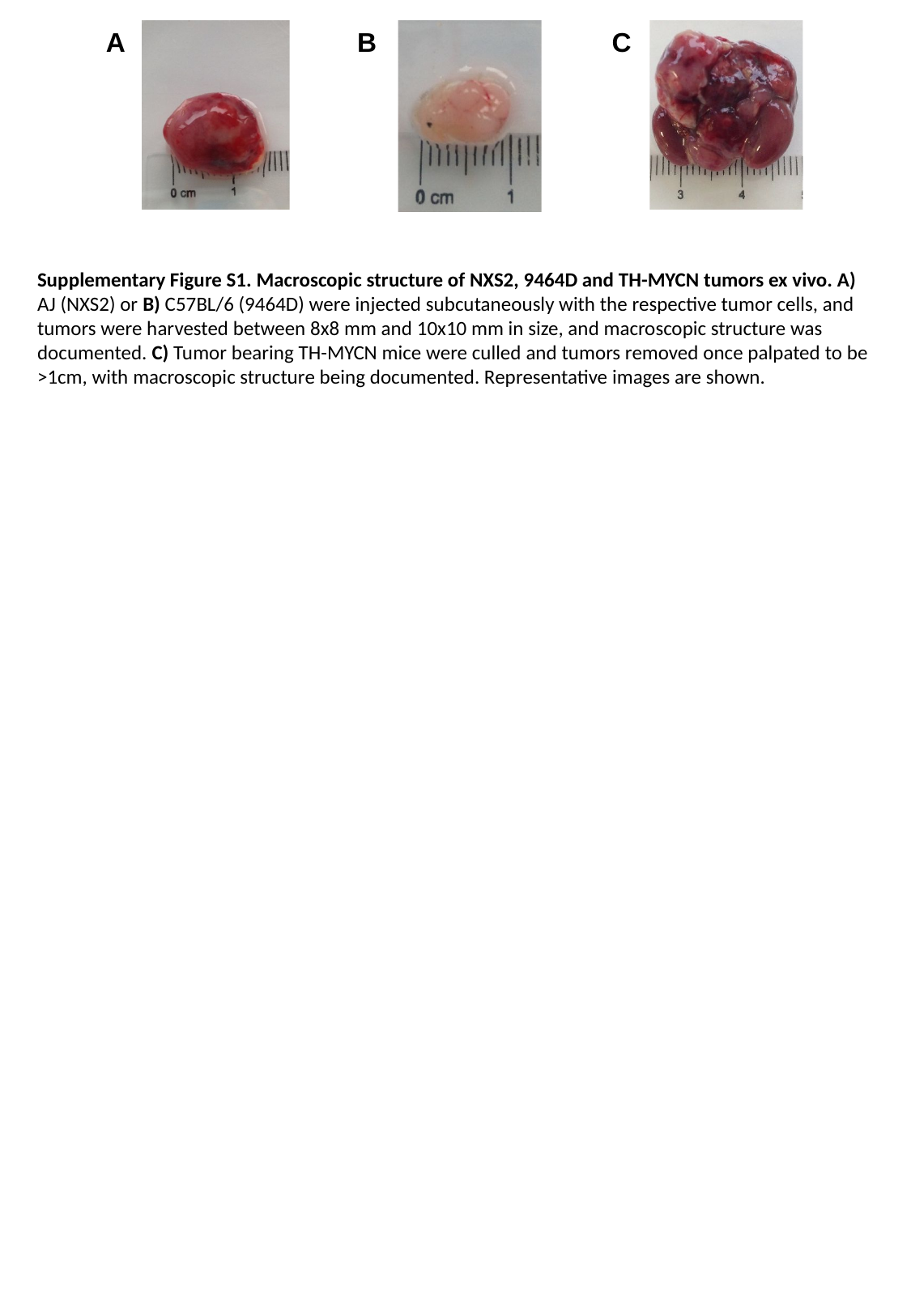

A
C
B
Supplementary Figure S1. Macroscopic structure of NXS2, 9464D and TH-MYCN tumors ex vivo. A) AJ (NXS2) or B) C57BL/6 (9464D) were injected subcutaneously with the respective tumor cells, and tumors were harvested between 8x8 mm and 10x10 mm in size, and macroscopic structure was documented. C) Tumor bearing TH-MYCN mice were culled and tumors removed once palpated to be >1cm, with macroscopic structure being documented. Representative images are shown.

## Slide 4
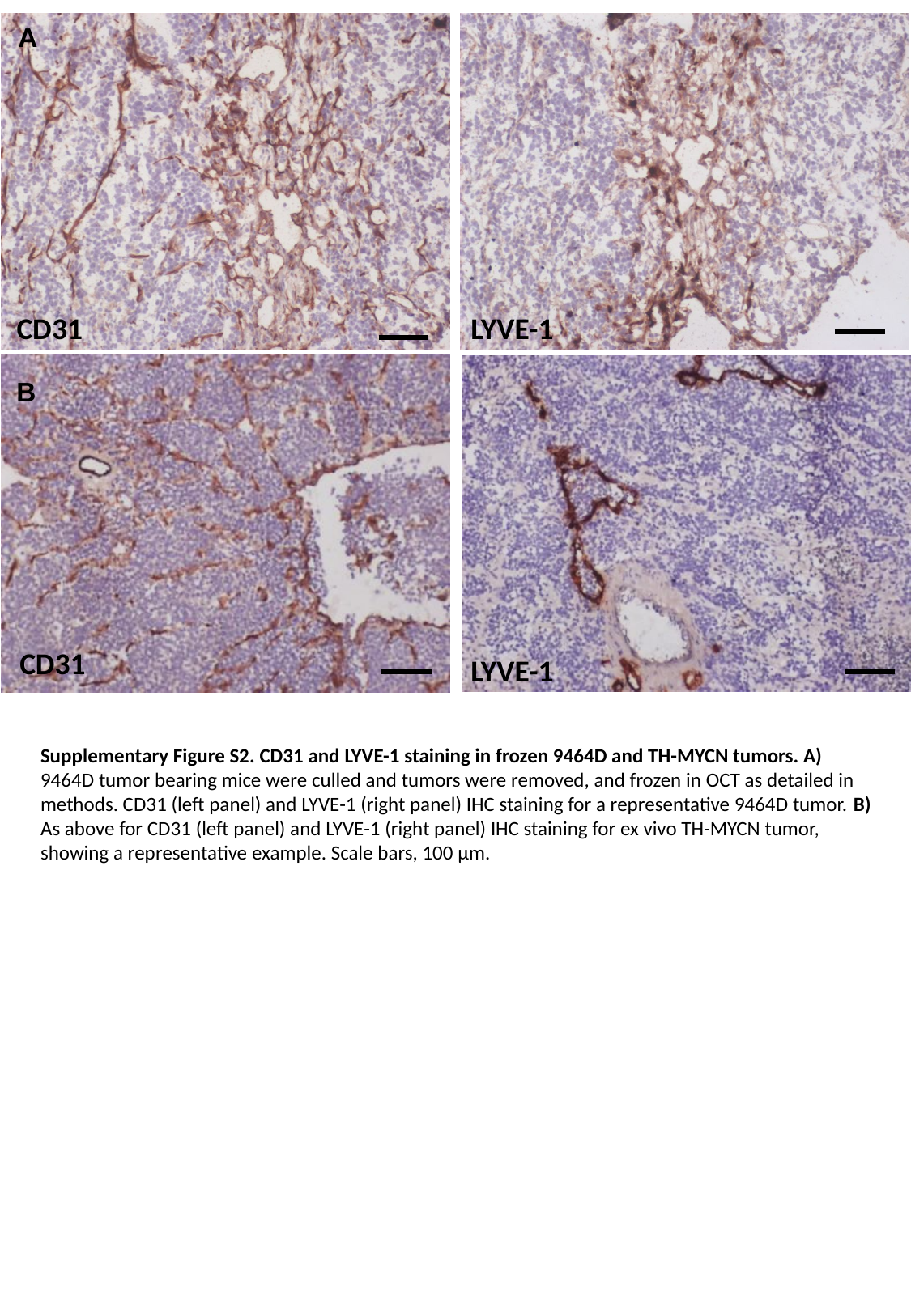

CD31
A
B
CD31
LYVE-1
LYVE-1
Supplementary Figure S2. CD31 and LYVE-1 staining in frozen 9464D and TH-MYCN tumors. A) 9464D tumor bearing mice were culled and tumors were removed, and frozen in OCT as detailed in methods. CD31 (left panel) and LYVE-1 (right panel) IHC staining for a representative 9464D tumor. B) As above for CD31 (left panel) and LYVE-1 (right panel) IHC staining for ex vivo TH-MYCN tumor, showing a representative example. Scale bars, 100 µm.

## Slide 5
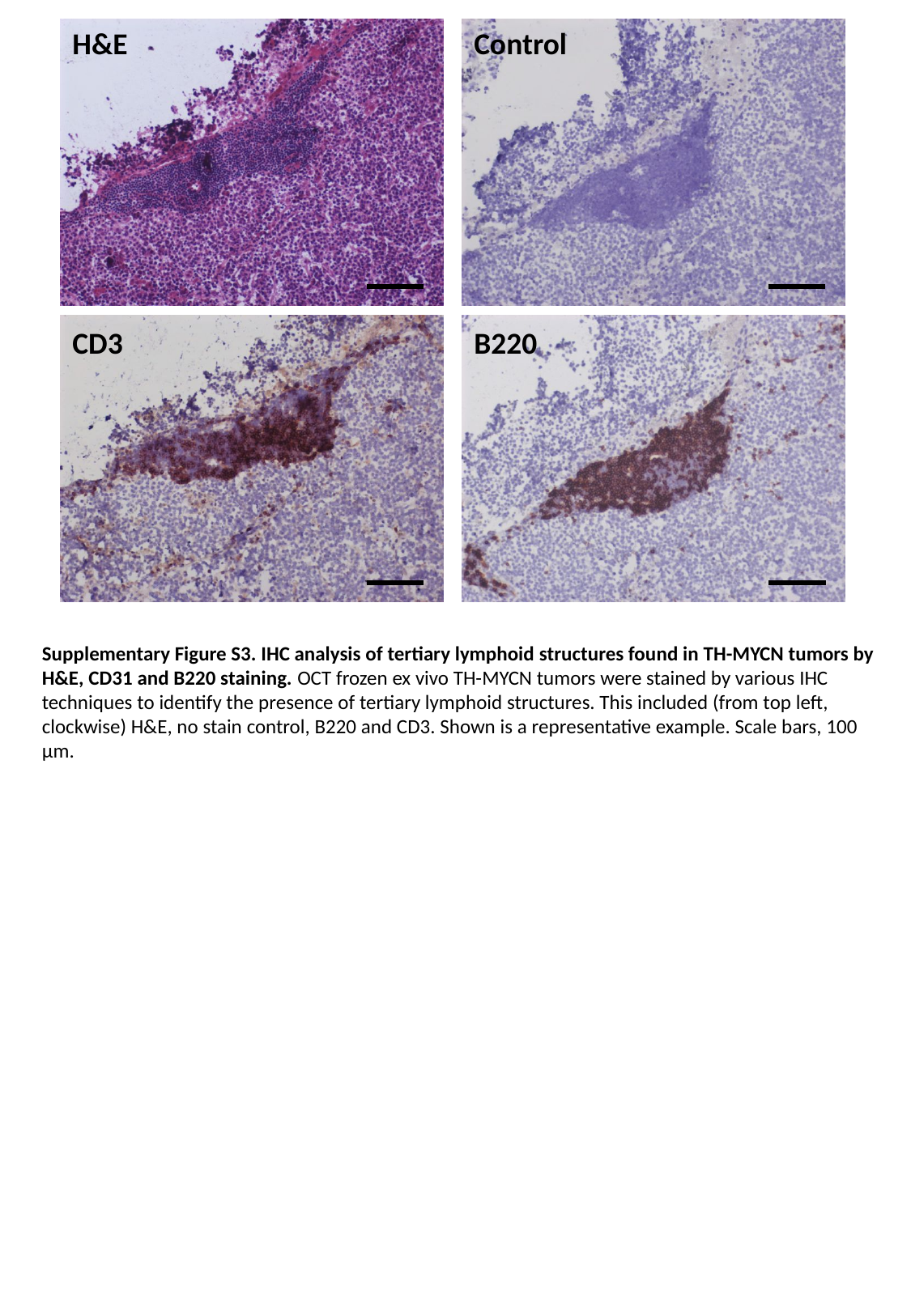

H&E
Control
B220
CD3
Supplementary Figure S3. IHC analysis of tertiary lymphoid structures found in TH-MYCN tumors by H&E, CD31 and B220 staining. OCT frozen ex vivo TH-MYCN tumors were stained by various IHC techniques to identify the presence of tertiary lymphoid structures. This included (from top left, clockwise) H&E, no stain control, B220 and CD3. Shown is a representative example. Scale bars, 100 µm.

## Slide 6
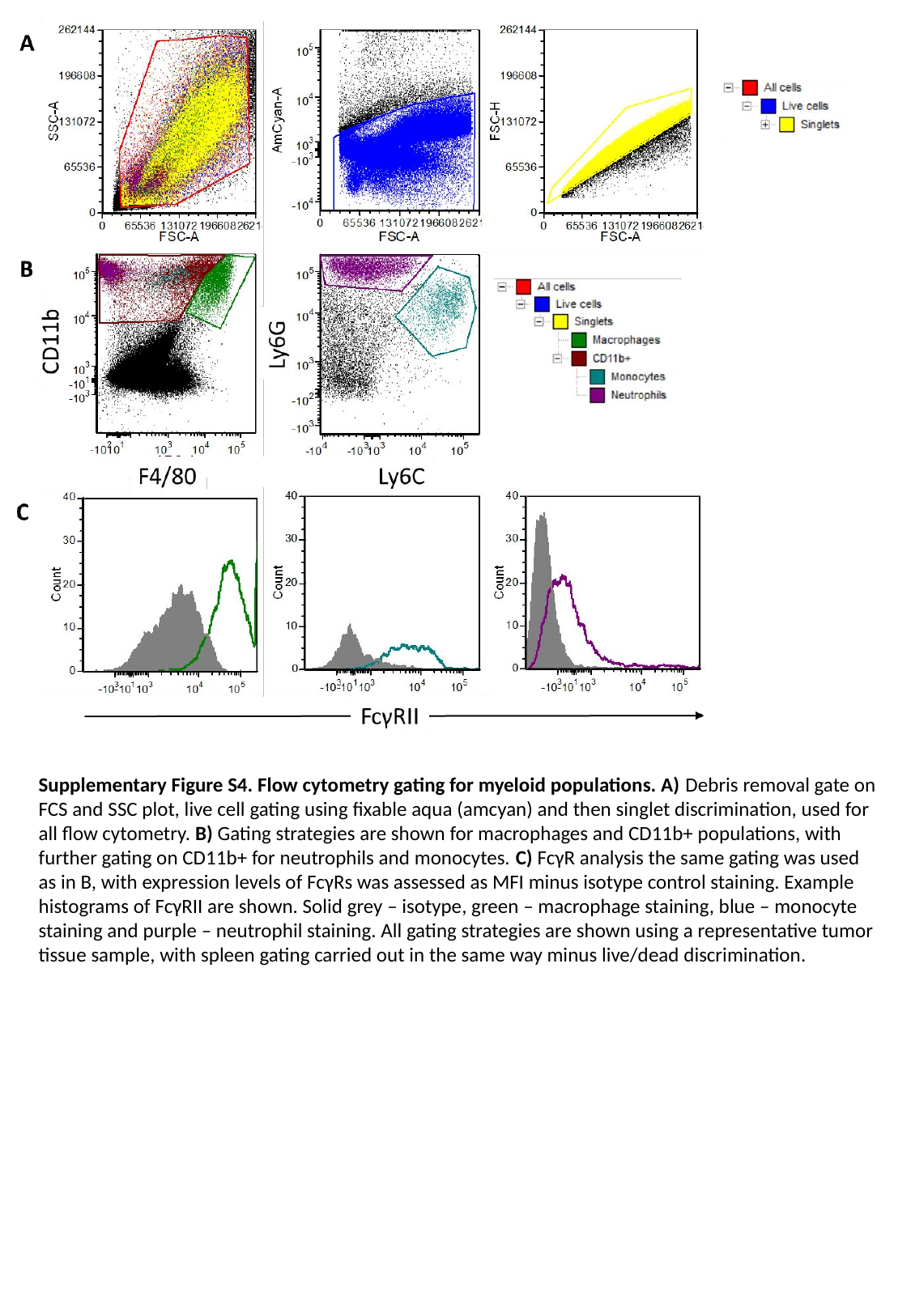

Supplementary Figure S4. Flow cytometry gating for myeloid populations. A) Debris removal gate on FCS and SSC plot, live cell gating using fixable aqua (amcyan) and then singlet discrimination, used for all flow cytometry. B) Gating strategies are shown for macrophages and CD11b+ populations, with further gating on CD11b+ for neutrophils and monocytes. C) FcγR analysis the same gating was used as in B, with expression levels of FcγRs was assessed as MFI minus isotype control staining. Example histograms of FcγRII are shown. Solid grey – isotype, green – macrophage staining, blue – monocyte staining and purple – neutrophil staining. All gating strategies are shown using a representative tumor tissue sample, with spleen gating carried out in the same way minus live/dead discrimination.

## Slide 7
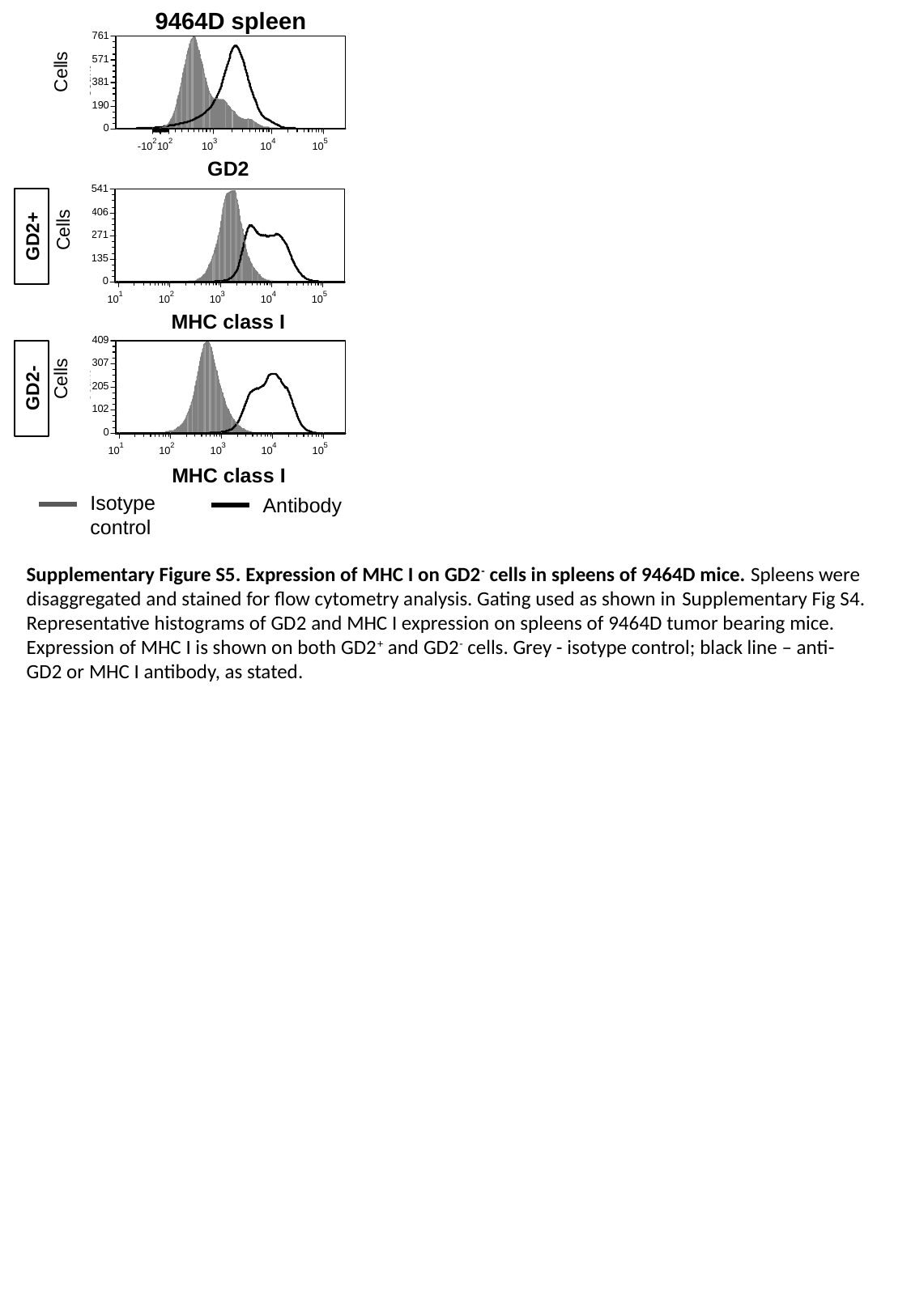

9464D spleen
Cells
GD2
Cells
GD2+
MHC class I
Cells
GD2-
MHC class I
Isotype control
Antibody
Supplementary Figure S5. Expression of MHC I on GD2- cells in spleens of 9464D mice. Spleens were disaggregated and stained for flow cytometry analysis. Gating used as shown in Supplementary Fig S4. Representative histograms of GD2 and MHC I expression on spleens of 9464D tumor bearing mice. Expression of MHC I is shown on both GD2+ and GD2- cells. Grey - isotype control; black line – anti-GD2 or MHC I antibody, as stated.

## Slide 8
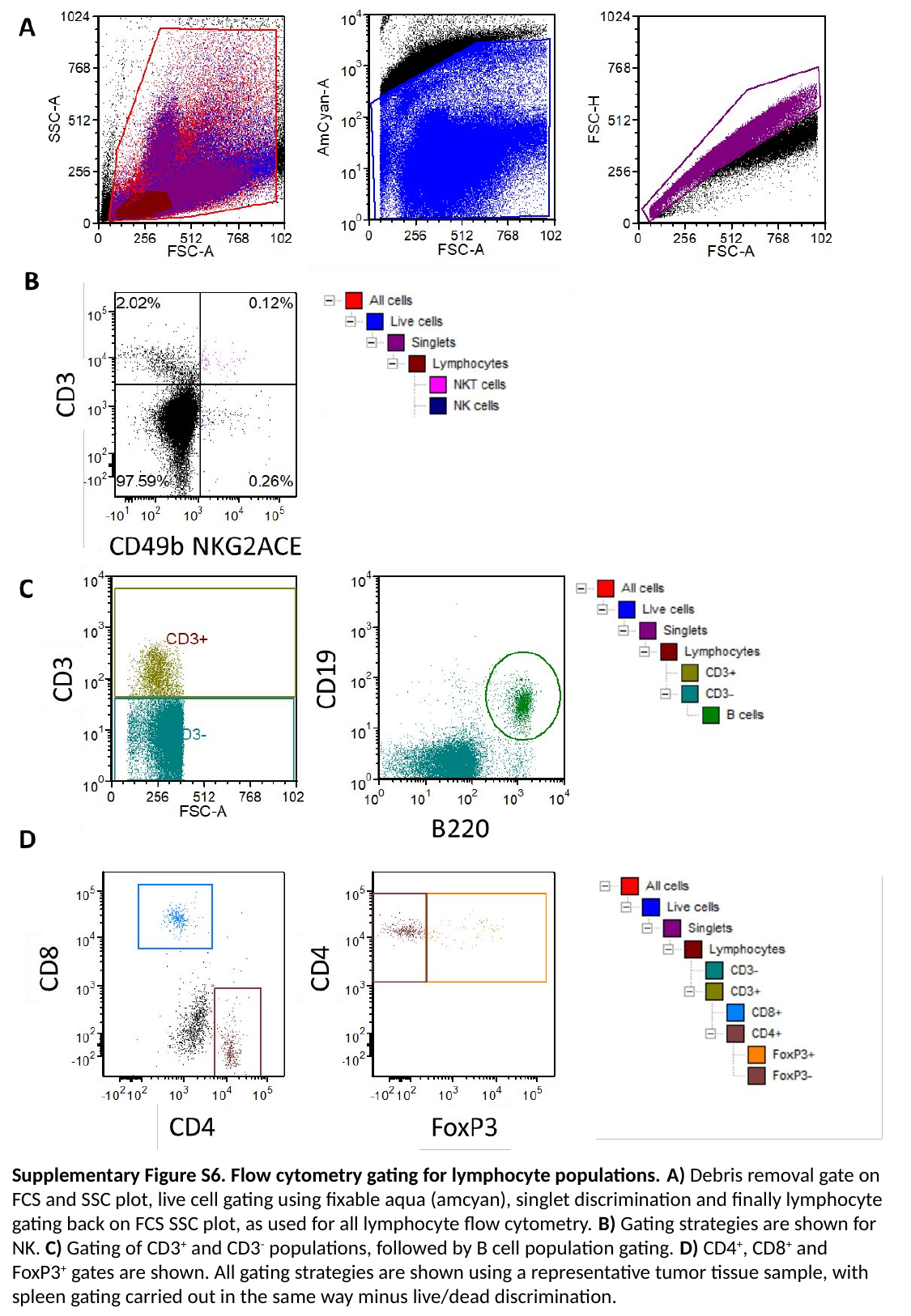

Supplementary Figure S6. Flow cytometry gating for lymphocyte populations. A) Debris removal gate on FCS and SSC plot, live cell gating using fixable aqua (amcyan), singlet discrimination and finally lymphocyte gating back on FCS SSC plot, as used for all lymphocyte flow cytometry. B) Gating strategies are shown for NK. C) Gating of CD3+ and CD3- populations, followed by B cell population gating. D) CD4+, CD8+ and FoxP3+ gates are shown. All gating strategies are shown using a representative tumor tissue sample, with spleen gating carried out in the same way minus live/dead discrimination.
